# ﻿Different, but still the same: integrative taxonomy confirms a new species of *Eresus* Walckenaer, 1805 (Araneae, Eresidae) from the South Caucasus

**DOI:** 10.3897/zookeys.1249.159081

**Published:** 2025-08-12

**Authors:** Alireza Zamani, Armen Seropian, Noushig Zarikian, Natalia Bulbulashvili, Tamás Szűts

**Affiliations:** 1 Department of Biology, University of Turku, Turku, Finland University of Turku Turku Finland; 2 Caucasus Leibniz Biodiversity Research Center (LBiC), Ilia State University, 3/5 Cholokashvili Ave., Tbilisi 0179, Georgia. Ilia State University Tbilisi Georgia; 3 Scientific Center of Zoology and Hydroecology, National Academy of Sciences, Yerevan, Armenia Scientific Center of Zoology and Hydroecology, National Academy of Sciences Yerevan Armenia; 4 Rustaveli st. 9, 1400, Gori, Georgia Unaffiliated Gori Georgia; 5 Department of Zoology, University of Veterinary Medicine Budapest, Budapest, Hungary University of Veterinary Medicine Budapest Budapest Hungary

**Keywords:** Biodiversity hotspot, integrative taxonomy, ladybird spiders, Transcaucasia

## Abstract

This study represents the first step toward a systematic revision of the velvet spider genus *Eresus* Walckenaer, 1805 in the Caucasus. Here, *Eresustranscaucasicus* sp. nov. is described using an integrative approach, based on male specimens collected from the South Caucasus. The species was previously reported as *E.kollari* Rossi, 1846 from Armenia and as *Eresus* sp. from Georgia. Intraspecific variations in both coloration patterns and conductor shape, which have been rarely documented in this genus, are illustrated. The validity of previous records of *E.kollari* in the region is discussed.

## ﻿Introduction

Eresidae C.L. Koch, 1845 is a relatively small family of spiders, currently comprising 116 valid species in nine genera and two subfamilies. Commonly known as velvet spiders, they are primarily distributed throughout the Palaearctic and Afrotropical regions. A few species of *Stegodyphus* Simon, 1873 occur in the Oriental region, and one species, *S.manaus* Kraus & Kraus, 1992, is known from Brazil (WSC 2025; Zamani et al. 2025).

The type genus, *Eresus* Walckenaer, 1805, consists of 32 accepted species and five subspecies distributed from the Mediterranean Basin to Korea and the Russian Far East (WSC 2025; Zamani et al. 2025). Males of most *Eresus* species exhibit vibrant coloration patterns, earning them the common name “ladybird spiders.” The taxonomy and diversity of *Eresus* remain poorly explored across much of its range. Seventeen species have been described in the 21^st^ century, six based solely on morphological characteristics (Zamani et al. 2020; Lin et al. 2021; Řezáč et al. 2023; Al-Yacoub et al. 2025; Lecigne et al. 2025) and eleven using an integrative approach (Řezáč et al. 2008; Kovács et al. 2015; Lin et al. 2022; Zamani et al. 2025). The latter approach is particularly important given the high degree of interspecific uniformity in the copulatory structures within the genus.

The Caucasus, with the highest biodiversity of any temperate forest region worldwide and one of two biodiversity hotspots in West Asia (Zazanashvili and Mallon 2009), remains particularly understudied regarding the diversity of ladybird spiders. Currently, only three *Eresus* species are known from this region (Otto 2022; Mikhailov 2024): *E.kollari* Rossi, 1846, the most widely distributed species of the genus, at least based on the current state of knowledge; *E.lavrosii* Mcheidze, 1997, a black-and-white-colored species known from Georgia (Mcheidze 1997; Seropian et al. 2025), Turkey (Zamani et al. 2020; Kara et al. 2025), Armenia (Kalashian et al. 2023; Zarikian et al. 2023), and Azerbaijan (Seropian et al. 2025); and an unidentified species from Georgia that could not be matched with any known species based on either COI barcode sequences or morphology (Seropian et al. 2023).

The present study represents the first step toward a systematic revision of *Eresus* in the Caucasus. Here, we describe a new species that was initially reported as *E.kollari* from Armenia by Zarikian (2022) and as *Eresus* sp. from Georgia by Seropian et al. (2023), using an integrative approach and based on material collected from both countries.

## ﻿Materials and methods

### ﻿Morphological examination and the map

**Figure 1.**
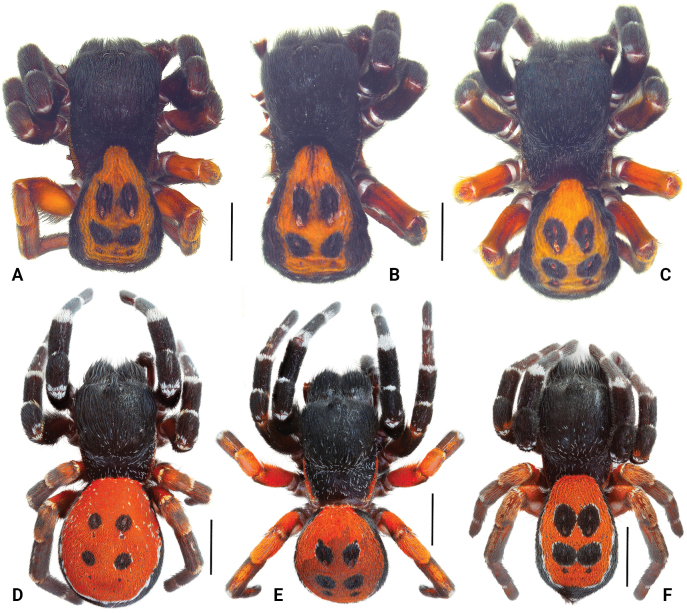
Habitus of the male holotype (**B.**ZMUT 1017, from Armenia) and paratypes (**A, C.**HNHM 11678 and ZMUT 1018, from Armenia; **D–F.**ISU-CaBOL 1009989, 1009988, 1018701, from Georgia) of *Eresustranscaucasicus* sp. nov., dorsal view. Scale bars: 4 mm.

**Figure 2.**
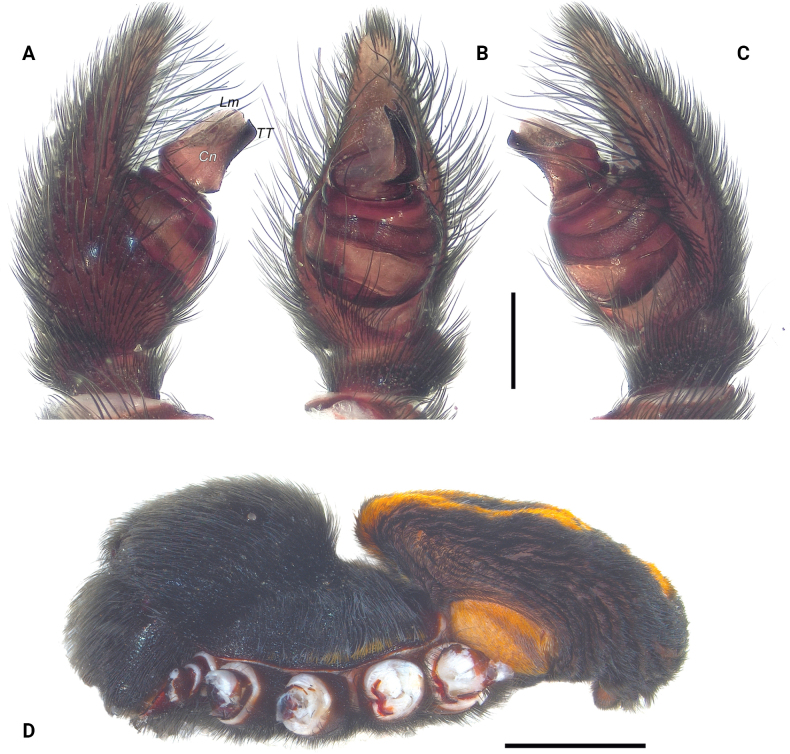
Palp (**A–C**) and habitus (**D**) of the male holotype (ZMUT 1017, from Armenia) of *Eresustranscaucasicus* sp. nov. **A.** Prolateral view; **B.** Ventral view; **C.** Retrolateral view; **D.** Lateral view. Abbreviations: Cn – conductor, Lm – lamella, TT – terminal tooth. Scale bars: 0.4 mm (**A–C**), 4 mm (**D**).

**Figure 3.**
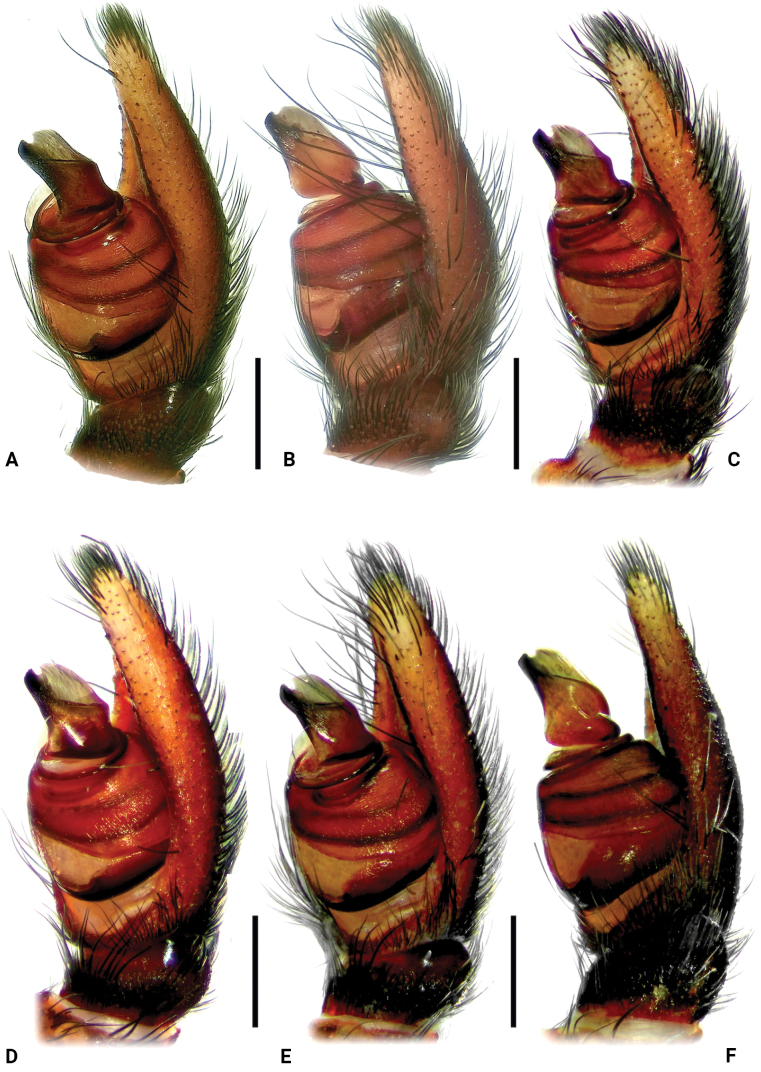
Palps of the male paratypes of *Eresustranscaucasicus* sp. nov., retrolateral view. **A, B.**HNHM 11448 and HNHM 11678, respectively, from Armenia; **C–F.**ISU-CaBOL 1009988, 1009989, 1018701, 1018700, respectively, from Georgia. Scale bars: 0.4 mm.

**Figure 4.**
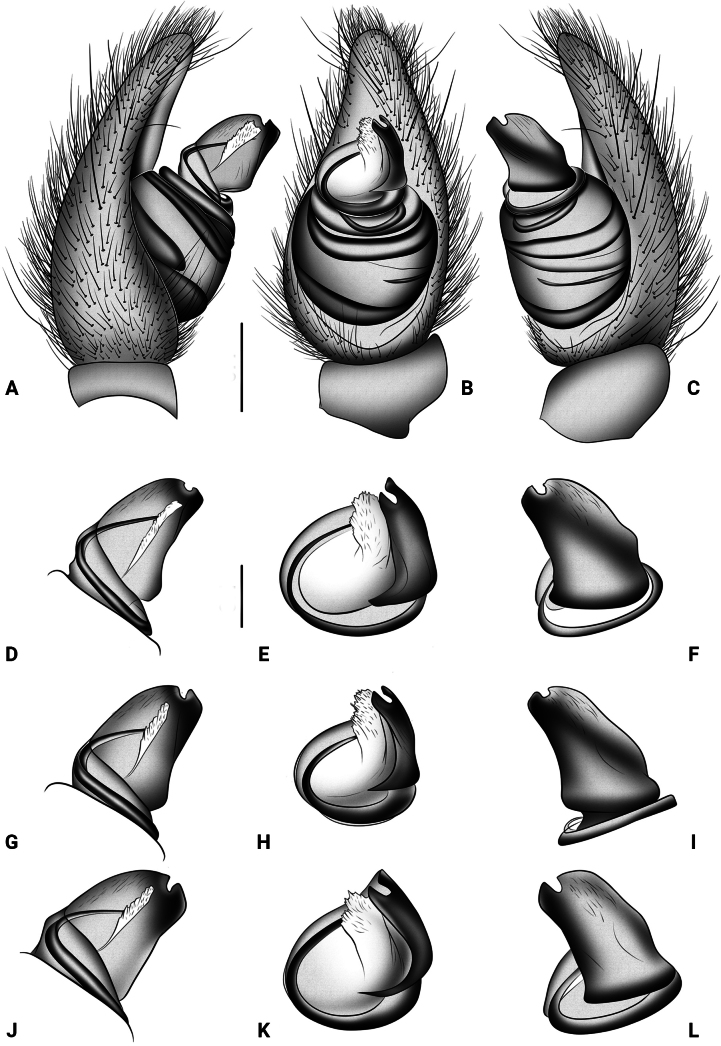
Palps of the male paratypes (**A–C.**ISU-CaBOL 1009988; **D–F.**ISU-CaBOL 1009989; **G–I.**ISU-CaBOL 1018700; **J–L.**ISU-CaBOL 1018701) of *Eresustranscaucasicus* sp. nov. from Georgia. **A–C.** Tibia, cymbium and bulb, prolateral, ventral and retrolateral views; **D, G, J.** Embolic division, prolateral view; **E, H, K.** Same, ventral view; **F, I, L.** Same, retrolateral view. Scale bars: 0.4 mm (**A–C**), 0.1 mm (**D–L**).

The Armenian specimens (Figs 1A–C, 2A–D, 3A, B) were examined at the Department of Zoology, University of Veterinary Medicine Budapest (Hungary), with the left palp detached and setae shaved off using a lancet. Specimens were fixed in position on sand for acquiring the habitus images, and in commercial hand sanitizer for the palp images. Multifocal images were compiled using Helicon Focus 7.0, licensed to TS. Stack images of different focal planes for the habitus were captured using a Tucsen MiChrome digital camera attached to a Nikon SMZ1000 stereomicroscope, while palp images were taken by a Tucsen TrueChrome Metrics digital camera on a Nikon Eclipse E200 compound microscope. The Georgian specimens (Figs 1D–F, 3C–F, 4A–L) were examined at the Institute of Ecology, Ilia State University (Georgia), using a Zeiss Stemi 508 Stereo Microscope with 8:1 Zoom and a Zeiss Apo 1.5× FWD 53 mm front lens attached. Drawings were made based on multifocal microscope photographs stacked in Zerene Stacker 1.04, using a Wacom CTH-690 Intuos Medium Pen and Touch Tablet with the programs Krita v. 2.9.7 and Photoshop CS6 v. 13.0 licensed to AS. Drawings show the left palp. All measurements are provided in millimeters and were taken from images. Leg segments were measured on the dorsal side and are listed as: total (femur, patella, tibia, metatarsus, tarsus). The distribution map (Fig. 6) was created using SimpleMappr (Shorthouse 2010).

### ﻿Depositories

**HNHM**Hungarian Natural History Museum, Budapest, Hungary (E. Lazányi);

**ISU** Ilia State University, Tbilisi, Georgia;

**ZMUT** Zoological Museum of the University of Turku, Turku, Finland (V. Vahtera).

### ﻿Molecular procedures, phylogenetic analysis, and species delimitation

DNA from the Armenian specimens was extracted from a single leg using standard extraction kits. PCR amplification was conducted at the Department of Zoology, University of Veterinary Medicine Budapest, using the LCO1490/HCO2198 primers (Folmer et al. 1994). Capillary electrophoresis was performed with an ABI 3500xL sequencer (Applied Biosystems, Foster City, CA, USA) through the commercial services of Biomi (Gödöllő, Hungary).

DNA extraction from the Georgian specimens at ISU followed a customized protocol (Seropian et al. 2023). The extracted DNA and remaining specimen material were deposited in the scientific collections of ISU, and the resulting sequences were submitted to the Barcode of Life Data Systems (BOLD) database.

Phylogenetic analysis was conducted using IQ-TREE 2.0 (Minh et al. 2020) with the command “-m TESTNEW -bb 1000 -alrt 1000.” The resulting tree was edited in FigTree (Rambaut 2018).

Species delimitations were performed using various methods: ABGD (Puillandre et al. 2012), ASAP (Puillandre et al. 2021), and PTP (Zhang et al. 2013). The results were saved in SPART format (Miralles et al. 2022) and compared using LIMES (Ducasse et al. 2020).

## ﻿Results

**Figure 5.**
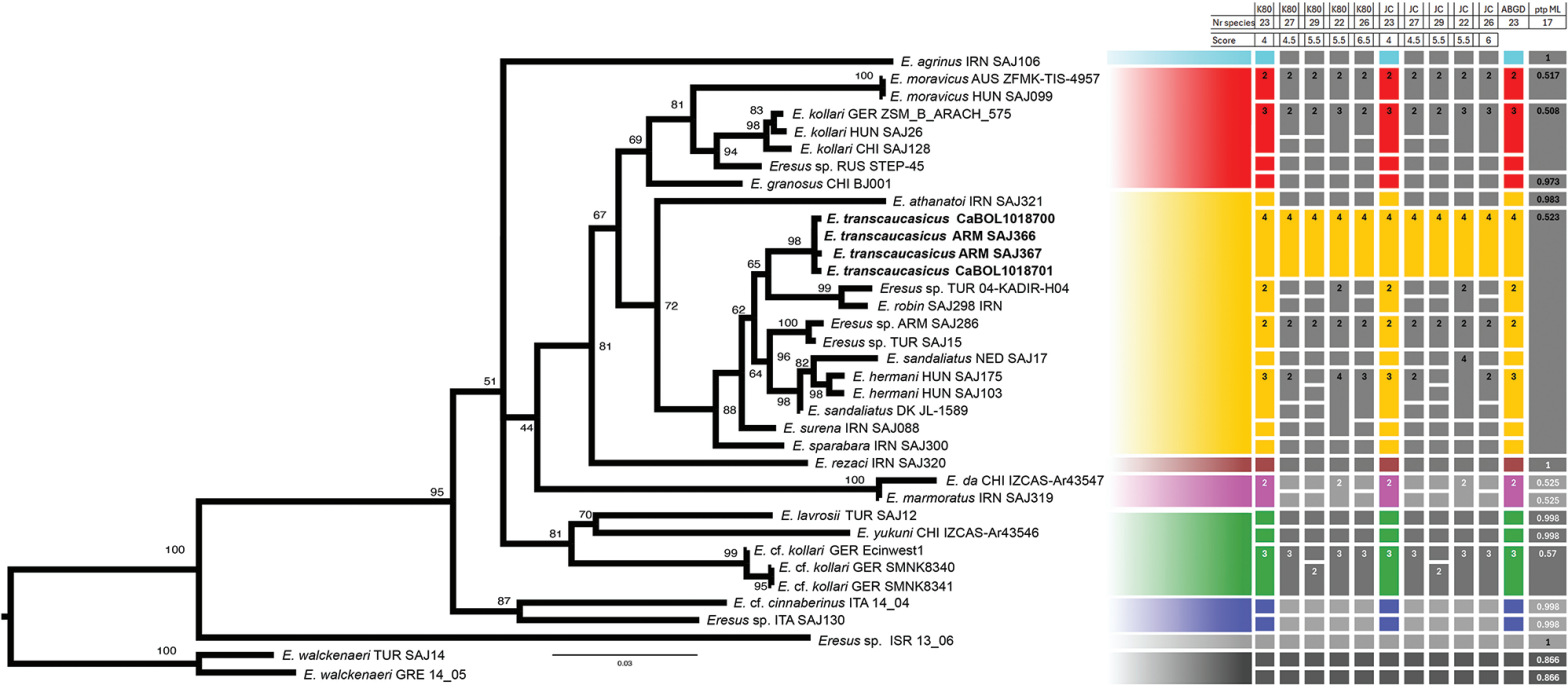
Phylogenetic and species delimitation results, based on the COI dataset: the maximum likelihood phylogram, along with the results of various species delimitation methods. Taxon names in bold represent the new species described here. Morphology-based species concepts are indicated by their respective Latin names. Each horizontal bar represents a putative species as inferred by a molecular method. The preferred delimitation results are highlighted in color, others are in grayscale. Different colors correspond to recognized species groups as explained in Zamani et al. (2025).

The phylogenetic analysis resulted in a tree (Fig. 5) that is mostly congruent with that of Zamani et al. (2025), with one exception: *E.surena* Zamani & Szűts, 2025 is placed basal in the *sandaliatus* group instead of sister to the group consisting of *E.sandaliatus* (Martini & Goeze, 1778) and *E.hermani* Kovács, Prazsák, Eichardt, Vári & Gyurkovics, 2015. *Eresustranscaucasicus* sp. nov. is placed sister to a clade comprising *E.robin* Zamani & Szűts, 2025 and *Eresus* sp. from Batman, Turkey.

**Figure 6.**
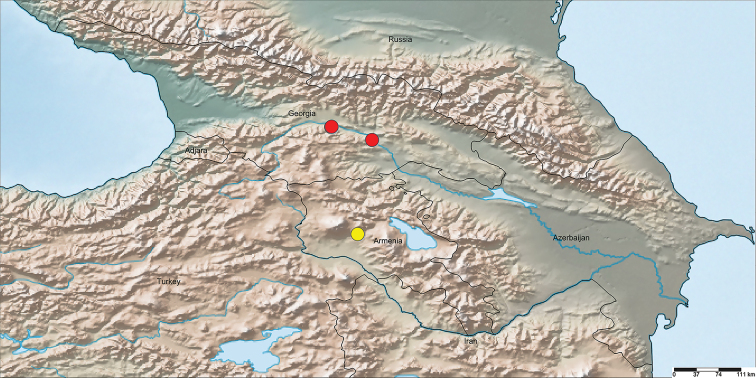
Collection localities of the type material of *Eresustranscaucasicus* sp. nov. The yellow circle indicates the type locality in Armenia; the red circles indicate the collection localities of the paratypes from Georgia.

Among the species delimitation methods, the ASAP (Assemble Species by Automatic Partitioning) analysis under both the K80 and Jukes-Cantor substitution models produced the same groupings with the lowest ASAP score. This was identical to the ABGD delimitation of 23 species. Alternative groupings based on suboptimal ASAP scores ranged from 22 to 29 species (see Fig. 5B for details). However, in all these groupings, the four representatives of *Eresustranscaucasicus* sp. nov. were consistently treated as a single species.

Among the twelve groupings generated by the four delimitation methods, eleven recovered the four vouchers of *E.transcaucasicus* sp. nov. as a single species. The exception was PTP, which included it within a larger cluster of species of the *sandaliatus* group (i.e., *E.hermani*; *E.robin*; *E.sandaliatus*; *E.sparabara* Zamani & Szűts, 2025; *E.surena*; *Eresus* sp.). These results support the conspecificity of specimens from the two geographically distant localities in Armenia and Georgia. For details see Suppl. material 1.

### ﻿Taxonomy

**Family Eresidae C.L. Koch, 1845**

#### ﻿Genus Eresus

Taxon classificationAnimaliaAraneaeEresidae

Walckenaer, 1805

58B99E50-4BC3-5255-BC94-B52DF88431BE

##### Type species.

*Araneacinnaberina* Olivier, 1789; by subsequent designation (Thorell 1870: 200).

#### ﻿ Eresus transcaucasicus sp. nov.

Taxon classificationAnimaliaAraneaeEresidae

88BAEDA8-82A9-5197-A63B-448DC044026E

https://zoobank.org/9613F024-904B-4F8E-9102-8EFCB62F4A76

Figs 1A–F , 2A–D , 3A–F , 4A–L

Eresus kollari : Zarikian 2022: 759, fig. 1 (♂).
Eresus sp.: Seropian et al. 2023: 237, fig. S1 (♂).

##### Type material.

***Holotype*** • ♂ (ZMUT 1017, SAJ366): Armenia: Aragatsotn Province: Ara Mountains, 40°23'N, 44°28'E, 1770 m, 03.11.2022, leg. N. Zarikian. ***Paratypes***: • 5♂ (ZMUT 1018, HNHM 11678, HNHM 11448, HNHM 11589, HNHM 11491), collected with the holotype; • 2♂ (ISU; CaBOL-IDs 1018700, 1018701): Georgia: Shida Qartli Region: Gori, path to Tsedisi Fortress, steppe, 41°58'02.6"N, 44°05'54.2"E, 850 m, 23.10.2021, leg. A. Seropian, N. Bulbulashvili; • 1♂ (ISU; CaBOL-ID 1009989): Gori, steppe, 41°58'31.1"N, 44°06'03.6"E, 600 m, 25.09.2024, leg. N. Bulbulashvili; • 1♂ (ISU; CaBOL-ID 1009988): Tbilisi: Dighomi village, heathland, 41°46'49.8"N, 44°42'14.0"E, 667 m, 28.09.2024, leg. A. Seropian, N. Bulbulashvili.

##### Diagnosis.

The new species belongs to the *sandaliatus* group sensu Zamani et al. (2025). In the overall shape of the conductor (Cn) and the general coloration pattern of the body and appendages, it most closely resembles *E.kollari* and *E.hermani*. It can be distinguished from *E.kollari* by its more robust terminal tooth (TT) having a distinct bulge (Figs 2C, F, I, L, 3C, F cf. Miller et al. 2012: fig. 43J). From *E.hermani*, it differs in the conductor distinctly longer than wide in retrolateral view (*vs.* almost as long as wide; Figs 2C, F, I, L, 3C, F cf. Kovács et al. 2015: fig. 3B).

##### Description.

***Male*** (holotype). Habitus as in Figs 1B, 2D. Total length 8.21; carapace 4.68 long, 3.21 wide, 2.50 high; abdomen 4.78 long, 3.76 wide. Carapace and chelicerae dark brown, almost black, densely coated with black setae; pars cephalica elevated, covered with black setae; pars thoracica with sparse white setae, margins with few orange setae. Palpal segments without white setae; femur, patella and tibia densely covered with dark setae of black or grey tone. Legs I and II densely coated with black setae, with rings of white setae on proximal and distal joints of segments; legs III and IV coated with red setae coverage; distal end of tibiae and metatarsi with white ring of setae. Abdomen black on sides, anterior part of dorsum and posterior to epigastric fold; dorsum red, with two pairs of large and one pair of small black dots; venter anterior to epigastric fold red; booklung covers with short red setae. Measurements of leg segments: I: 9.02 (2.89, 1.45, 1.51, 1.86, 1.31); II: 7.75 (2.45, 1.37, 1.31, 1.53, 1.09); III: 6.60 (2.19, 1.23, 1.06, 1.30, 0.82); IV: 9.09 (2.82, 1.62, 1.85, 1.72, 1.08).

Palp as in Fig. 2A–C; conductor (Cn) distinctly longer than wide; terminal tooth (TT) well-developed and robust, slightly bulging, subequal to lamella (Lm), with smooth transition to base of conductor in lateral view; lamella >2× longer than high; lamellar groove shallow.

Variation (*n* = 5). Habitus of paratypes from Armenia as in Fig. 1A, C, and from Georgia as in Fig. 1D–F. Measurements: total length 8.21–8.92; carapace length 4.68–4.72, width 3.21–3.35. Sparse white setae may be present at black spots around sigilla (Fig. 1D–F). Palp of paratypes from Armenia as in Fig. 3A, B, and from Georgia as in Figs 3C–F, 4A–L. Conductor: length/width ratio: 1.2–1.5; very slight shoulder present in some specimens (Figs 2C, 4C, F); depth of lamellar groove slightly varying (Figs 3A–F, 4C, F, I, L).

***Female*.** Unknown.

##### Habitat.

The specimens were collected under stones and on the ground in warm, dry mountain slopes with sparse vegetation.

##### Phenology.

Early to late autumn.

##### Distribution.

Known only from the listed localities in Lesser Caucasus Mountains in Armenia and Georgia (Fig. 5).

##### Etymology.

The specific epithet refers to the distribution of the species in the South Caucasus, also known as Transcaucasia.

## ﻿Discussion

Traditionally, *Eresus* species have been separated based on coloration and/or characters of the copulatory organs. The former’s usefulness for species delimitation “is constrained by its intraspecific variability,” whereas the latter exhibits a “high degree of shape uniformity” and is therefore considered “not as useful for discrimination” (Řezáč et al. 2008: 280, 267). Here, we introduce DNA barcoding as a new line of evidence for species delimitation and demonstrate a high degree of intraspecific color variation (Fig. 1), a factor rarely tested in taxonomic studies. Regarding palpal characters, we also illustrate considerable intraspecific variation (Figs 2–4), in contrast to the conclusions of Řezáč et al. (2008).

However, we must also note two important considerations. First, our species delimitation was based on a single maternally inherited gene, which has its limitations (Dellicour and Flot 2018). Second, our dataset included many singletons, which may affect the resulting groupings. Nonetheless, we firmly believe that such exploratory work will facilitate further integrative research on this genus.

The reproductive periods, and consequently the surface activity of males, are phenologically separated among *Eresus* species. Broadly, they can be categorized into two groups: spring-early summer and late summer-autumn, with the latter including the species newly described herein. Based on previous records from the Caucasus (Azerbaijan, Dagestan, and Georgia; Otto 2022), it is likely that at least one additional species belonging to the *sandaliatus* group exists in the region, phenologically distinct from the one described here, as nearly all mature males have been collected in spring or early summer. Unfortunately, almost none of the authors have provided diagnostic drawings or photographs of the copulatory organs. Mcheidze (1997) reported several locations for *E.kollari* in Georgia (Shiraki, Kasristskali, Vashlovani Reserve); however, the absence of collection data and the poor quality of illustrations prevent a definitive assignment of these records to a particular group. Similarly, records of this species from Azerbaijan by Dunin (1984, 1988; as *E.niger* (Petagna, 1787)) and Marusik et al. (2005; as *E.cinnaberinus* (Olivier, 1789)) were considered doubtful by Marusik et al. (2005: 138): “Judging from the vulva, our specimen may belong to another sibling species”.

## Supplementary Material

XML Treatment for Eresus

XML Treatment for Eresus transcaucasicus

## ﻿Additional information

### Use of AI

No use of AI was reported.

### Author ORCIDs

Alireza Zamani https://orcid.org/0000-0002-8084-9666

Armen Seropian https://orcid.org/0000-0003-3777-9954

Noushig Zarikian https://orcid.org/0000-0001-8334-8413

Tamás Szűts https://orcid.org/0000-0001-8954-0641

### Data availability

All of the data that support the findings of this study are available in the main text or Supplementary Information.

## Supplementary materials

Supplementary material 1 Sequence alignment used in the study Data type pdf https://binary.pensoft.net/file/1393279This dataset is made available under the Open Database License (http://opendatacommons.org/licenses/odbl/1.0/). The Open Database License (ODbL) is a license agreement intended to allow users to freely share, modify, and use this Dataset while maintaining this same freedom for others, provided that the original source and author(s) are credited.Alireza Zamani, Armen Seropian, Noushig Zarikian, Natalia Bulbulashvili, Tamás Szűts
