# Supplementary material for: ﻿Different, but still the same: integrative taxonomy confirms a new species of Eresus Walckenaer, 1805 (Araneae, Eresidae) from the South Caucasus
Source: Zookeys. 2025 Aug 12;1249:1–13. doi: 10.3897/zookeys.1249.159081 (PMC12365673; doi:10.3897/zookeys.1249.159081)
Supplement: Supplementary material 1 — Sequence alignment used in the study [file zookeys-1249-001_article-159081__-s001.pdf]

S1: Sequence alignment used in the study

>AR451-10_HQ982805_BATMAN_TUR

NNCATTGTATTTAATTTTTGGAGCTTGATCAGCTATAGTTGGGACTTCAATAAGAATAATTATTCGAACG
GAACTAGGACAATCTGGGAGATTATTAGGGGATGATCATTTGTATAATGTTGTTGTTACTGCTCATGCT
TTTGTGATAATTTTTTTTATAGTTATACCAATTATGATTGGGGGGTTTGGAAACTGATTAGTTCCTTTAATA
TTAGGGGCGCCTGATATAGCTTTTCCTCGAATAAATAATTTGAGTTTTTGGTTATTACCTCCTTCATTGTT
TATGTTATTTATATCTTCTATAGTAGAAATGGGTGTAGGAACAGGGTGAACTGTTTATCCTCCTTTAGCT
TCATTAATAGGTCATGCGGGGAGATCAGTAGATTTTGCTATTTTTTCATTACATTTAGCTGGGGCTTCTT
CTATTATAGGGGCTATTAATTTTATTACGACTGTAATTAATATACGTTCATATGGGATGACTATAGAGAA
GGTACCGTTATTTGTTTGATCTGTATTAATTACTGCAGTGTTATTATTGTTATCTTTACCTGTTTTAGCAGG
TGCTATTACAATATTATTAACTGATCGAAATTTTAATACATCGTTTTTTGACCCTGCTGGGGGAGGAGAT
CCTATTTTATTTCAACATTTATTT

>BJ001_*E. granosus*

NNCATTGTATTTAATTTTTGGAGCTTGATCAGCTATAGTTGGAACTTCAATAAGAATAATTATTCGAACG
GAATTAGGACAATCTGGGAGATTATTAGGAGATGATCATTTGTATAATGTTGTTGTTACTGCTCATGCTT
TTGTAATAATTTTTTTTATAGTTATGCCAATTATAATTGGTGGGTTTGGGAATTGATTGGTTCCTTTAATATT
AGGAGCACCTGATATAGCTTTTCCTCGAATAAATAATTTGAGTTTTTGGTTATTGCCTCCTTCATTGTTTA
TATTATTTATATCTTCTATAGTAGAAATGGGTGTAGGGACAGGGTGAACTGTTTACCCTCCTTTAGCCT
CATTAATAGGTCATGCGGGGAGATCAGTAGATTTTGCTATTTTTTCATTACATTTAGCTGGGGCTTCTTC
TATTATGGGGGCTATTAATTTTATTACTACTGTACTTAATATACGTTCATATGGAATAACTATAGAGAAGG
TACCATTATTTGTTTGATCTGTTTTAATTACTGCGGTATTATTATTATTATCTTTACCTGTTTTAGCGGGTG
CTATTACAATATTATTAACTGATCGAAATTTTAATACATCGTTTTTTGATCCTGCTGGGGGAGGAGATCC
NNNNNNNNNNNNNNNNNNNNNN

>*E. transcaucasicus* CaBOL1018700_CaBOL_1018700

GACATTGTATTTAATTTTTGGAGCTTGATCAGCTATAGTTGGGACTTCAATAAGAATAATTATTCGGACG
GAATTAGGACAATCTGGGAGATTATTGGGGGATGATCATTTGTATAATGTTGTTGTTACTGCTCATGCT
TTTGTGATAATTTTTTTTATAGTTATACCAATTATGATTGGGGGGTTTGGGAATTGATTAGTTCCTTTAATG
TTGGGGGCGCCTGATATAGCTTTTCCTCGAATAAATAATTTGAGTTTTTGGTTATTACCCCCTTCATTAT
TTATGTTATTTATATCTTCTATAGTAGAAATGGGTGTAGGAACGGGGTGAACTGTGTATCCTCCTTTAGC
CTCATTAATAGGTCATGCGGGGAGATCAGTAGATTTCGCTATTTTTTCATTACATTTAGCTGGGGCTTC
TTCTATTATAGGGGCTATTAATTTTATTACAACTGTAATTAATATACGTTCATATGGGATGACTATAGAGA
AGGTGCCGTTATTTGTTTGATCTGTATTAATTACTGCGGTGTTATTGTTGTTATCTTTACCTGTTTTAGCG
GGTGCTATTACAATATTGTTAACTGATCGAAATTTTAATACATCGTTTTTTGACCCTGCTGGGGGAGGA
GATCCTATTTTATTTCAACATTTATTT

>*E._transcaucasicus*_CaBOL_1018701

GACATTGTATTTAATTTTTGGAGCTTGATCAGCTATAGTTGGGACTTCAATAAGAATAATTATTCGGACG
GAATTAGGACAATCTGGGAGATTATTGGGGGATGATCATTTGTATAATGTTGTTGTTACTGCTCATGCT
TTTGTGATAATTTTTTTTATAGTTATACCAATTATGATTGGGGGGTTTGGGAATTGATTAGTTCCTTTAATG
TTGGGGGCGCCTGATATAGCTTTTCCTCGAATAAATAATTTGAGTTTTTGGTTATTACCCCCTTCATTAT
TTATGTTATTTATATCTTCTATAGTAGAAATGGGTGTAGGAACAGGGTGAACTGTGTATCCTCCTTTAGC
CTCATTAATAGGTCATGCGGGGAGATCAGTGGATTTCGCTATTTTTTCATTACATTTAGCTGGGGCTTC
TTCTATTATAGGGGCTATTAATTTTATTACAACTGTAATTAATATACGTTCATATGGGATGACTATAGAGA
AGGTGCCGTTATTTGTTTGATCTGTATTAATTACTGCGGTGTTATTGTTGTTATCTTTACCTGTTTTAGCG

GGTGCTATTACAATATTGTTAACTGATCGAAATTTTAATACATCGTTTTTTGACCCTGCTGGGGGAGGA
GATCCTATTTTATTTCAACATTTATTT

>DQ973153_*E.cinnaberinus*_Ecinwest1_GER

NNNNNNNNNNNNNNNNNNNNNNNNNNNNNNNNNNNNNNNNNNNNNNNNNNNNNNNN
NNNNNNNNNNNNNNNNNNNNNNNNNNNNNNNNNNNNNNNNNNNNNNNNNNNNNNNN
NNNNNNNNNNNNNNNNNNNNNNNNNNNNNNNNNNNNNNNNNNNNNNNNNNNNNNNN
NNNNNNNNNNNNNNNNNNNNNNNNNNNNNNNNNNNNNNNNNNNNNNNNNNNNNNNN
NNNNNNNNNNNNNNNNNNNNNNNNNNNNNNNNNNNNNNCCTTCATTGTTTATATTATTTATGT
CTTCTATGGTAGAGATGGGTGTAGGAACAGGGTGAACTGTTTATCCTCCTTTGGCTTCATTAATAGGC
CATTCGGGAAGATCAGTGGATTTTGCTATTTTTTCATTACATTTAGCTGGGGCTTCTTCTATTATAGGAG
CTATTAATTTCATTACAACTGTAATTAATATACGTTCATATGGAATAACTATGGAGAAGGTACCATTATTT
GTTTGGTCTGTATTAATTACTGCAGTATTATTATTACTATCTTTACCTGTTTTAGCAGGTGCTATTACAATA
TTATTAACTGATCGAAATTTTAATACATCGTTTTTTGACCCTGCTGGGGGAGGGGACCCGGTTTTATTT
CAACATTTATTT

>FJ948997.1_E.sp._13_06_ISR

NNNNNNNNNNNNNNNNNNNNNNNNNNNNNNNNNNNNNNNNNNNNNNNNNNNNNNNN
NNNNNNNNNNNNNNNNNNNNNNNNNNNNNNNNNNNNNNNNNNNNNNNNNNNNNNNN
NNNNNNNNNNNNNNNNNNNNNNNNNNNNNNNNNNNNNNNNNNNNNNNNNNNNNNNN
NNNNNNNNNGTTTGGGAATTGATTAGTACCGTTAATGTTAGGAGCTCCTGATATAGCATTTCCTCGA
ATAAATAATTTAAGATTTTGATTATTACCTCCTTCATTGTTTATATTGTTTATATCTTCTATAGTAGAGATAG
GTGTAGGAACTGGATGAACTGTATATCCTCCTTTGGCTTCATTAATAGGACATGCTGGAAGATCAGTG
GATTTTGCTATTTTTTCGTTACATTTGGCTGGTGCTTCTTCTATTATAGGAGCTATTAATTTTATTACAACT
GTAATTAATATACGTTCTTATGGAATGACTATGGAAAGGGTGCCGTTATTTGTTTGATCTGTATTAATTAC
TGCTGTATTATTATTATTATCTTTACCAGTGTTAGCGGGGGCTATTACAATGTTGTTGACTGATCGAAATT
TTAATACGTCATTTTTTGACCCTGCTGGGGGAGGTGATCCAATTTTATTTCAACATTTGTTT

>FJ948998.1_*E.cf._kollari*_14_04_ITA

NNNNNNNNNNNNNNNNNNNNNNNNNNNNNNNNNNNNNNNNNNNNNNNNNNNNNNNN
NNNNNNNNNNNNNNNNNNNNNNNNNNNNNNNNNNNNNNNNNNNNNNNNNNNNNNNN
NNNNNNNNNNNNNNNNNNNNNNNNNNNNNNNNNNNNNNNNNNNNNNNNNNNNNNNN
NNNNNNNNNATTCGGGAATTGGTTGGTTCCTTTAATATTAGGGGCACCTGATATAGCTTTCCCTCGA
ATGAATAATTTAAGTTTTTGGTTATTACCTCCTTCATTGTTTATGTTATTTATATCTTCTATAGTGGAAATGG
GTGTAGGAACAGGATGAACTGTATATCCTCCTTTAGCTTCATTAATAGGTCATGCGGGGAGATCGGT
GGATTTTGCTATTTTTTCATTACATTTAGCTGGAGCTTCTTCTATTATAGGAGCTATTAATTTTATTACAAC
TGTAATTAATATACGTTCATATGGAATAACTATGGAAAAAGTGCCATTATTTGTTTGATCTGTATTAATTA
CTGCGGTATTGTTATTACTATCTTTACCTGTTCTGGCAGGTGCTATTACAATATTATTAACTGATCGAAA
TTTTAATACGTCATTTTTTGATCCTGCTGGAGGAGGAGATCCAGTTTTATTTCAGCATTTATTT

>FJ948999.1_*E.walckenaeri*_14_5_GRE

NNNNNNNNNNNNNNNNNNNNNNNNNNNNNNNNNNNNNNNNNNNNNNNNNNNNNNNN
NNNNNNNNNNNNNNNNNNNNNNNNNNNNNNNNNNNNNNNNNNNNNNNNNNNNNNNN
NNNNNNNNNNNNNNNNNNNNNNNNNNNNNNNNNNNNNNNNNNNNNNNNNNNNNNNN
NNNNNNNNNGTTTGGGAATTGGTTAGTACCTTTAATATTAGGGGCTCCTGATATAGCTTTTCCTCGAA
TGAATAATTTAAGTTTTTGGTTATTACCTCCTTCATTATTTATATTATTTATATCTTCTATAGTAGAGATGGG
TGTGGGAACTGGATGAACTGTATATCCTCCTTTAGCATCGTTAATAGGTCATGCAGGAAGATCAGTG

GATTTTGCTATTTTTTCATTACATTTAGCTGGGGCTTCATCTATTATAGGGGCTATCAATTTTATTACAACT
GTAATCAACATACGTTCATATGGAATAACAATAGAGAGGGTGCCATTATTTGTTTGATCTGTGTTGATTA
CTGCAGTATTATTATTATTATCTTTACCTGTATTAGCCGGTGCAATTACAATATTATTAACTGATCGAAAT
TTTAATACTTCATTTTTTGACCCTGCTGGAGGGGGAGATCCAATTTTATTTCAACATCTATTT

>KX443582.1_*E.sandaliatus*_DK

NNNNNNNNNNNNNNNNNNNNNNNNNNNNNNNNNNNNNNNNNNNNNNNNNNNNNNNN
NNNNNNNNNNNNNNNNNNNNNNNNNNNNNNNNNNNNNNNNNNNNNNNNNNNNNNNN
NNNNNNNNNNNNNNNNNNNNNNNNNNNNNNNNNNNNNNNNNNNNNNNNNNNNNNNN
NNNNNNNNNGTTTGGGAATTGATTGGTTCCTTTAATGTTGGGGGCACCTGATATAGCTTTTCCTCGA
ATAAATAATTTGAGTTTTTGGTTATTACCCCCTTCATTGTTTATGTTATTTATATCTTCTATAGTAGAAATGG
GTGTAGGAACAGGGTGAACTGTATATCCTCCTTTAGCCTCATTAATAGGTCATGCGGGGAGATCAGT
AGATTTCGCTATTTTTTCATTACATTTAGCTGGAGCTTCTTCTATTATAGGGGCTATTAATTTTATTACAAC
TGTAATTAATATACGTTCATATGGGATGACTATGGAGAAGGTACCGTTATTTGTTTGATCTGTATTAATTA
CTGCGGTATTATTATTGTTATCTTTACCTGTTTTAGCAGGTGCTATTACAATATTGTTNNNNNNNNNNN
NNNNNNNNNNNNNNNNNNNNNNNNNNNNNNNNNNNNNNNNNNNNNNNNNNNNNNNN
NNNNNNN

>KX537083_*E.cinnaberinus*_GER

NNCGTTGTATTTAATTTTTGGAGCTTGATCAGCTATAGTTGGAACTTCAATAAGAATAATTATTCGAACG
GAATTAGGACAATCTGGGAGATTATTAGGAGATGATCATTTGTATAATGTTGTTGTTACTGCTCATGCTT
TTGTTATAATTTTTTTTATAGTTATACCAATTATAATTGGAGGGTTTGGGAATTGATTGGTTCCTTTAATGTT
AGGGGCACCTGATATAGCTTTTCCTCGAATAAATAATTTAAGTTTTTGGTTATTACCTCCTTCATTGTTTA
TATTATTTATGTCTTCTATAGTAGAAATGGGTGTAGGGACAGGGTGAACTGTTTATCCTCCTTTAGCATC
ATTAATAGGTCATGCGGGGAGATCAGTAGATTTTGCTATTTTTTCACTACATTTAGCTGGGGCTTCTTCT
ATTATGGGGGCTATTAATTTTATTACAACTGTAATTAATATACGTTCATATGGAATAACAATGGAGAAGG
TATCATTATTTGTTTGATCTGTTTTAATTACTGCGGTATTATTGTTGTTATCTTTACCTGTTTTAGCGGGCG
CTATTACAATATTATTAACTGATCGAAATTTTAATACATCGTTTTTTGACCCTGCTGGGGGAGGAGATC
CTATTTTATTTCAACATTTATTT

>KY268507.1_*E.moravicus*_AUS

NNCATTATATTTAATTTTTGGAGCTTGATCAGCTATAGTAGGAACTTCAATAAGAATAATTATTCGAACA
GAATTAGGGCAATCTGGGAGATTATTAGGAGATGATCATTTGTATAATGTTGTTGTTACTGCCCATGCT
TTTGTGATAATTTTTTTTATAGTCATACCAATTATGATTGGGGGGTTTGGTAATTGATTAGTTCCTTTAATG
TTAGGGGCACCTGATATAGCTTTTCCTCGAATAAATAATTTGAGTTTTTGGTTATTACCTCCTTCATTATT
TATATTATTTATATCTTCTATAGTAGAAATGGGTGTAGGGACAGGGTGAACTGTTTATCCTCCTTTAGCG
TCGTTAATAGGTCATGCGGGGAGATCAGTAGATTTTGCTATTTTTTCATTACATTTAGCTGGGGCTTCTT
CTATTATGGGAGCTATTAATTTTATTACAACTGTAATTAATATACGTTCATATGGAATGACAATGGAGAA
GGTACCGTTATTTGTTTGATCTGTATTAATTACTGCAGTATTGTTATTGTTATCTTTACCTGTTTTAGCAGG
TGCTATTACAATGTTGTTAACTGATCGAAATTTTAATACATCGTTTTTTGACCCTGCTGGGGGAGGAGA
CCCAATTTTATTTCAACATTTATTT

>OL352218.1_*E.moravicus*_SAJ099

NNCATTATATTTAATTTTTGGAGCTTGATCAGCTATAGTAGGAACTTCAATAAGAATAATTATTCGAACA
GAATTAGGGCAATCTGGGAGATTATTAGGAGATGATCATTTGTATAATGTTGTTGTTACTGCCCATGCT
TTTGTGATAATTTTTTTTATAGTCATACCAATTATGATTGGGGGGTTTGGTAATTGATTAGTTCCTTTAATG
TTAGGGGCACCTGATATAGCTTTTCCTCGAATAAATAATTTGAGTTTTTGGTTATTACCTCCTTCATTATT

TATATTATTTATATCTTCTATAGTAGAAATGGGTGTAGGGACAGGGTGAACTGTTTATCCTCCTTTAGCG
TCGTTAATAGGTCATGCGGGGAGATCAGTAGATTTTGCTATTTTTTCATTACATTTAGCTGGGGCTTCTT
CTATTATGGGAGCTATTAATTTTATTACAACTGTAATTAATATACGTTCATATGGAATGACAATGGAGAA
GGTACCGTTATTTGTTTGATCTGTATTAATTACTGCAGTATTGTTATTGTTATCTTTACCTGTTTTAGCAGG
TGCTATTACAATGTTGTTAACTGATCGAAATTTTAATACATCGTTTTTTGACCCTGCTGGGGGAGGAGA
CCCAATTTTATTTCAACATTTATTT

>OL352220.1_*E.hermani*_SAJ103

NNCATTGTATTTAATTTTTGGAGCTTGATCAGCTATAGTTGGGACTTCGATAAGAATAATTATTCGAACG
GAATTAGGACAATCTGGGAGATTATTAGGGGATGATCATTTGTATAATGTTGTTGTTACTGCTCATGCTT
TTGTGATAATTTTTTTTATAGTTATACCAATTATAATTGGGGGGTTTGGGAATTGATTGGTTCCTTTAATATT
GGGGGCACCTGATATAGCTTTTCCTCGAATAAATAATTTGAGTTTTTGGTTATTACCCCCTTCATTGTTT
ATGTTATTTATATCTTCTATAGTAGAAATAGGTGTAGGAACAGGGTGAACTGTATATCCTCCTTTAGCTT
CATTAATAGGTCATGCGGGGAGATCAGTAGATTTCGCTATTTTTTCATTACATTTAGCTGGAGCTTCTT
CTATTATAGGGGCTATTAATTTTATTACAACTGTAATTAATATACGTTCATATGGGATGACTATGGAGAA
GGTACCGTTATTTGTTTGATCTGTATTAATTACTGCGGTATTATTATTGTTATCTTTACCTGTTTTAGCAGG
TGCTATTACAATGTTGTTAACTGATCGAAATTTTAATACATCGTTTTTTGACCCTGCTGGGGGAGGAGA
TCCTATTTTATTTCAACATTTATTT

>OL352224.1_*E.lavrosiae*_SAJ12

NNCATTGTATTTAATTTTTGGAGCTTGATCAGCTATGGTTGGAACTTCAATAAGAATAATTATTCGAACA
GAATTAGGACAATCTGGGAGATTGTTAGGAGATGATCATTTGTATAATGTTGTTGTTACTGCTCATGCTT
TTGTAATGATTTTTTTTATAGTTATACCAATTTTAATTGGAGGGTTTGGGAATTGGTTAGTTCCTTTAATGTT
AGGGGCACCTGATATAGCTTTTCCTCGAATAAATAATTTGAGTTTTTGGTTATTACCCCCTTCATTGTTT
ATATTATTTATATCTTCTATAGTAGAGATAGGTGTAGGGACAGGGTGAACTGTTTATCCTCCTTTAGCCT
CATTAATAGGTCATGCGGGGAGATCAGTGGATTTTGCTATTTTTTCATTACATTTAGCAGGAGCTTCTT
CTATTATAGGAGCTATTAATTTTATTACAACTGTAATTAACATACGTTCGTATGGAATAACTATAGAGAG
GGTACCGTTATTTGTTTGGTCTGTGTTAATTACTGCAGTATTATTGTTATTATCTTTACCCGTTTTAGCAG
GTGCTATTACAATATTATTAACTGATCGTAATTTTAATACATCGTTTTTTGATCCTGCTGGAGGAGGGGA
TCCAGTTTTGTTTCAACATTTATTT

>OL352226.1_*E.kollari*_SAJ128_CHI

NNCGTTGTATTTAATTTTTGGAGCTTGATCAGCTATAGTTGGAACTTCAATAAGAATAATTATTCGAACG
GAATTAGGACAATCTGGGAGATTATTAGGAGATGATCATTTGTATAATGTTATTGTTACTGCTCATGCTT
TTGTTATAATTTTTTTTATAGTTATACCAATTATAATTGGAGGGTTTGGGAATTGATTGGTTCCTTTAATATT
AGGGGCACCTGATATAGCTTTTCCTCGAATAAATAATTTAAGTTTTTGGTTATTACCTCCTTCATTGTTTA
TATTATTTATGTCTTCTATAGTAGAAATGGGTGTAGGGACAGGGTGAACTGTTTATCCTCCTTTAGCATC
ATTAATAGGTCATGCGGGGAGATCAGTAGATTTTGCTATTTTTTCATTACATTTAGCTGGGGCTTCTTCT
ATTATGGGGGCTATTAATTTTATTACAACTGTAATTAATATACGTTCATATGGAATAACAATGGAGAAGG
TATCATTATTTGTTTGATCTGTTTTAATTACTGCGGTATTATTGTTGTTATCTTTACCTGTTTTAGCGGGTG
CTATTACAATATTATTAACTGATCGAAATTTTAATACATCGTTTTTTGATCCTGCTGGGGGAGGAGATCC
TATTTTATTTCAACATTTATTT

>OL352228.1_*E.sp.*_SAJ130_ITA

NNCGTTGTATTTAATTTTTGGAGCTTGGTCAGCTATAGTTGGAACTTCAATAAGAATAATTATTCGAACA
GAATTAGGGCAATCTGGGAGATTACTAGGAGATGATCATTTGTATAATGTTGTTGTTACAGCTCATGCT
TTTGTAATGATTTTTTTTATAGTTATACCAATTTTAATTGGAGGGTTTGGGAACTGGTTGGTTCCTTTAATA

TTGGGGGCGCCTGATATAGCTTTTCCTCGAATGAATAATTTGATTTTTTGGTTGTTACCTCCTTCATTGT
TTATGTTATTTATATCTTCTATAGTAGAAATGGGTGTAGGAACAGGGTGAACTGTATATCCTCCTTTAGC
TTCGTTAATAGGTCATGCGGGTAGATCGGTGGATTTTGCTATTTTTTCATTACATTTAGCTGGGGCTTCT
TCTATTATAGGAGCTATTAATTTTATTACAACTGTTATTAATATACGTTCATATGGAATAACTATGGAAAA
GGTACCGTTATTTGTTTGATCTGTACTAATTACTGCGGTATTGTTATTATTATCTTTACCTGTTCTAGCGG
GTGCTATTACGATATTATTAACTGATCGAAATTTTAATACATCATTTTTTGATCCTGCTGGGGGAGGAGA
TCCTGTTCTATTTCAACATTTATTT

>OL352229.1_*E.walckenaeri*_SAJ14_TUR

NNCGTTATATTTAATTTTTGGAGCTTGATCAGCTATAGTTGGGACTTCTATAAGAATGATTATTCGGACT
GAATTAGGGCAATCAGGGAGATTATTAGGGGATGATCATCTATATAATGTTATTGTTACTGCTCATGCT
TTTGTAATAATTTTTTTTATGGTTATGCCGATTTTGATTGGGGGATTTGGGAATTGGTTAGTACCTTTAATG
TTAGGGGCTCCTGATATAGCTTTTCCTCGAATGAATAATTTGAGTTTTTGGTTATTACCTCCTTCATTATT
TATGTTATTTATATCTTCTATAGTAGAGATAGGTGTGGGAACTGGATGAACTGTATATCCTCCTTTAGCG
TCGTTAATAGGTCATGCAGGAAGATCAGTAGATTTTGCTATTTTTTCATTACATTTAGCTGGGGCTTCAT
CTATTATAGGAGCTATCAATTTTATTACAACTGTAATTAATATACGTTCATATGGAATAACAATAGAGAG
GGTACCATTATTTGTTTGATCTGTGTTGATTACTGCAGTATTATTATTATTATCTTTACCTGTGTTAGCAG
GTGCAATTACAATATTATTGACTGATCGAAATTTTAATACTTCATTTTTTGACCCTGCTGGAGGGGGAG
ATCCAATTTTATTTCAGCATCTATTT

>OL352230.1_*Eresus*_sp_SAJ15

NNCATTGTATTTAATTTTTGGAGCTTGATCAGCTATAGTTGGGACTTCGATAAGAATAATTATTCGAACG
GAATTAGGACAATCTGGGAGATTATTAGGGGATGATCATTTGTATAATGTTGTTGTTACTGCTCATGCTT
TTGTGATAATTTTTTTTATAGTTATGCCAATTATGATTGGGGGGTTTGGGAATTGATTAGTTCCTTTAATGT
TGGGGGCACCTGATATAGCTTTTCCTCGAATAAATAATTTGAGTTTTTGGTTATTACCCCCTTCATTGTT
TATGTTATTTATATCTTCTATAGTAGAAATGGGTGTCGGAACAGGGTGAACTGTGTATCCTCCTTTAGC
CTCATTAATAGGTCATGCGGGGAGATCAGTAGATTTCGCTATTTTTTCATTACATTTAGCTGGGGCTTC
TTCTATTATAGGGGCTATTAATTTTATTACAACTGTAATTAATATGCGTTCATATGGGATAACTATAGAGA
AGGTACCGTTATTTGTTTGATCTGTATTGATTACTGCGGTATTATTATTGTTATCTTTACCTGTTTTAGCAG
GTGCTATTACAATATTGTTAACTGACCGAAATTTTAATACATCGTTTTTTGACCCTGCTGGGGGAGGAG
ACCCTATTTTATTTCAACATTTATTT

>OL352231.1_*E.sandaliatus*_SAJ17_NED

NNCGTTATATTTAATTTTTGGAGCTTGATCAGCTATAGTTGGGACTTCGATAAGAATAATTATTCGAACG
GAATTAGGACAATCTGGGAGATTATTAGGGGATGATCATTTGTATAATGTTGTTGTTACTGCTCATGCTT
TTGTGATAATTTTTTTTATAGTTATACCAATTATGATTGGGGGGTTTGGGAATTGATTGGTTCCTTTAATAT
TGGGGGCACCTGATATAGCTTTTCCTCGAATAAATAATTTGAGTTTTTGGTTATTACCTCCTTCATTGTTT
ATGTTATTTTTATCTTCTATAGTAGAAATAGGTGTAGGAACAGGGTGAACTGTATATCCTCCTTTAGCCT
TATTAATAGGTCATGCGGGGAGATCAGTAGATTTCGTTATTTTTTCATTACATTTAGCTGGGGCTTCTTC
TATTATAGGGGCTATTAATTTTATTACAACTGTAATTAATATACGTTCATATGGGATGACTATGGAGAAG
GTACCGTTATTTGTTTGATCTGTATTAATTACTGCGGTATTATTATTATTATCTTTACCTGTTTTAGCAGGT
GCTATTACAATATTGTTAACTGATCGAAATTTTAATACATCGTTTTTTGACCCTGCTGGGGGAGGAGAC
CCTATTTTATTTCAACATTTATTT

>OL352232.1_*E.kollari*_SAJ26

NNCGTTGTATTTAATTTTTGGAGCTTGATCAGCTATAGTTGGAACTTCAATAAGAATAATTATTCGAACG
GAATTAGGGCAATCTGGGAGATTATTAGGAGATGATCATTTGTATAATGTTGTTGTTACTGCTCATGCTT

TTGTTATAATTTTTTTTATAGTTATACCAATTATAATTGGAGGGTTTGGGAATTGATTGGTTCCTTTAATGTT
AGGGGCACCTGATATAGCTTTTCCTCGAATAAATAATTTAAGTTTTTGGTTATTACCTCCTTCATTGTTTA
TATTATTTATGTCTTCTATAGTAGAAATGGGTGTAGGGACAGGGTGAACTGTTTATCCTCCTTTAGCGT
CATTAATAGGTCATGCGGGGAGATCAGTAGATTTTGCTATTTTTTCATTACATTTAGCTGGGGCTTCTTC
TATTATGGGGGCTATTAATTTTATTACAACTGTAATTAATATACGTTCATATGGAATAACAATGGAGAAG
GTATCATTATTTGTTTGATCTGTTTTAATTACTGCGGTATTATTGTTGTTATCTTTACCTGTTTTAGCGGGC
GCTATTACAATATTATTAACTGATCGAAATTTTAATACATCGTTTTTTGACCCTGCTGGGGGAGGAGAT
CCTATTTTATTTCAACATTTATTT

>OL352235.1_*E.cf._kollari*_SAJ38

NNCATTATATTTAATTTTTGGAGCTTGGTCGGCTATGGTTGGAACTTCAATAAGAATAATTATTCGAACA
GAATTAGGACAATCTGGGAGATTATTAGGAGATGATCATCTGTATAATGTTGTTGTTACTGCTCATGCTT
TTGTAATGATTTTTTTTATAGTTATACCAATTTTAATTGGAGGGTTTGGGAATTGATTAGTTCCTTTAATATT
AGGAGCACCTGATATAGCTTTTCCTCGAATAAATAATTTAAGTTTTTGGTTGTTACCTCCTTCATTGTTTA
TATTATTTATGTCTTCTATGGTAGAGATGGGTGTAGGAACAGGGTGAACTGTTTATCCTCCTTTGGCTT
CATTAATAGGCCATTCGGGAAGATCAGTGGATTTTGCTATTTTTTCATTACATTTAGCTGGGGCTTCTTC
TATTATAGGAGCTATTAATTTCATTACAACTGTAATTAATATACGTTCATATGGAATAACTATAGAGAAGG
TACCATTATTTGTTTGGTCTGTATTAATTACTGCAGTATTATTATTACTATCTTTACCTGTTTTAGCAGGTG
CTATTACAATATTATTAACTGATCGAAATTTTAATACATCGTTTTTTGACCCTGCTGGGGGAGGGGACC
CGATTTTATTTCAACATTTATTT

>OP376824.1_*E.da*_20220728_CHI

NNNNNNNNNNNNNNNNNNNNNNNNNNNGATCGGCTATAGTTGGGACTTCAATAAGAATAATTATT
CGAACAGAATTAGGACAGTCTGGAAGATTATTAGGAGATGATCATCTATATAATGTTGTTGTTACTGCT
CATGCTTTTGTAATAATTTTTTTTATAGTTATACCAATTTTAATTGGTGGATTTGGAAATTGGTTGGTCCCA
TTAATATTAGGAGCACCTGATATAGCTTTTCCTCGAATAAATAATTTAAGTTTTTGGTTGTTACCTCCTTC
TTTGTTTATATTATTTATATCTTCTATAGTAGAAATAGGTGTGGGGACAGGATGAACTGTGTATCCTCCTT
TAGCTTCGTTAATAGGTCATGCGGGGAGATCAGTAGATTTTGCTATTTTTTCATTACATTTAGCTGGGG
CTTCTTCTATTATAGGAGCTATTAATTTTATTACAACTGTAATTAATATACGTTCATATGGAATAACTATGG
AGAAGGTACCATTATTTGTTTGATCTGTATTAATTACTGCAGTGTTGTTATTATTATCTTTACCTGTTTTGG
CAGGTGCTATTACTATATTATTAACTGATCGAAATTTTAATACGTCGTTTTTTGATCCTGCTGGAGGGGG
GGATCCTATTTTATTTCAACACTTATTT

>OP434393.1_*E.yukuni*_YJL-2022b_20220914_CHI

NNNNNNNNNNNNNNNNNNNNNNNNNNNNNNCGGCTATAGTTGGAACTTCAATAAGAATAATTATT
CGAACAGAATTAGGACAATCTGGGAGATTGTTAGGAGATGACCATTTGTATAATGTTGTTGTTACTGCT
CATGCTTTTGTAATGATTTTTTTTATAGTTATGCCAATTTTAATTGGGGGGTTTGGGAATTGGTTAGTCCC
TCTAATATTAGGAGCACCTGATATAGCTTTTCCTCGAATGAATAATTTGAGTTTTTGGTTATTACCTCCTT
CATTGTTTATATTATTTATATCTTCTATGGTAGAGATGGGTGTAGGAACAGGATGAACTGTTTATCCTCC
TTTAGCTTCGTTAATAGGTCATGCAGGAAGATCAGTGGATTTTGCTATTTTTTCATTACATTTAGCTGGG
GCTTCCTCTATTATAGGAGCTATTAATTTTATTACAACTATTATTAATATGCGTTCTTATGGAATGAGTATG
GAGAAAGTACCATTATTTGTTTGGTCTGTATTAATTACTGCAGTATTATTATTATTATCTTTACCTGTTTTA
GCAGGAGCTATTACAATATTATTAACTGATCGTAATTTTAATACATCGTTTTTTGACCCTGCTGGGGGA
GGGGATCCAGTTTTATTTCANNNNNNNNNN

>SAJ088_*E.surena*_IRN

NNCATTGTATTTAGTTTTTGGAGCTTGATCAGCTATAGTTGGGACTTCAATAAGAATAATTATTCGAACG
GAATTAGGACAATCTGGGAGATTATTAGGAGATGATCATTTGTATAATGTTGTTGTTACTGCTCATGCTT
TTGTGATAATTTTTTTTATAGTTATACCAATTATGATTGGGGGGTTTGGGAATTGATTAGTTCCTTTAATGT
TGGGGGCACCTGATATAGCTTTTCCTCGAATAAATAATTTGAGTTTTTGGTTATTACCCCCTTCATTGTT
TATGTTATTTATATCTTCTATAGTAGAAATAGGTGTAGGAACAGGGTGAACTGTGTATCCTCCTTTAGCC
TCATTAATAGGTCATGCGGGGAGATCAGTAGATTTCGCTATTTTTTCATTACATTTAGCTGGGGCTTCTT
CTATTATAGGGGCTATTAATTTTATTACAACTGTAATTAATATACGTTCATATGGGATGACTATAGAAAAG
GTACCGTTATTTGTTTGATCTGTATTAATTACTGCGGTATTGTTATTGTTATCTTTACCTGTTTTAGCAGGT
GCTATTACAATATTGTTAACTGATCGAAATTTTAATACATCGTTTTTTGATCCTGCTGGGGGAGGAGNN
NNNNNNNNNNNNNNNNNNNNNNNNN

>SAJ106_*E.agrinus*_IRN

NNCATTGTATTTGATTTTTGGAGCTTGATCAGCTATAGTTGGAACTTCAATAAGAATAATTATTCGAACG
GAGTTAGGACAATCTGGGAGATTATTAGGGGATGATCATTTGTATAATGTTGTTGTTACTGCCCATGCT
TTTGTGATAATTTTTTTTATAGTTATACCTATTTTAATTGGAGGGTTTGGGAATTGATTGGTTCCTTTGATAT
TAGGGGCGCCCGATATGGCTTTTCCTCGGATAAATAATTTGAGTTTTTGGTTGTTGCCTCCTTCGTTGT
TTATATTATTTATGTCTTCTATAGTAGAAATAGGTGTAGGGACAGGATGAACTGTTTATCCCCCTTTAGC
TTCATTGATAGGTCATGAGGGAAGATCGGTGGATTTTGCTATTTTTTCATTACATTTAGCGGGGGCTTC
TTCTATTATAGGAGCTATTAATTTTATTACAACTGTAATTAATATACGTTCTTATGGAATAACTATAGAGAA
GGTGCCGTTATTTGTTTGATCTGTATTAATTACTGCAGTATTGTTATTATTGTCTTTGCCTGTATTAGCAG
GTGCTATCACAATATTATTAACTGATCGAAATTTTAATACATCGTTTTTTGATCCTGCTGGGGGAGGAG
ATCCAATTTTATTTCAACATTTATTT

>SAJ175_*E.hermani*_HUN

NNNNNNNNNNNNNNNNNNNNNNNNNNNNGATCAGCTATAGTTGGGACTTCGATAAGAATAATTATT
CGAACGGAATTAGGACAATCTGGGAGATTATTAGGGGATGATCATTTGTATAATGTTGTTGTTACTGCT
CATGCTTTTGTGATAATTTTTTTTATAGTTATACCAATTATAATTGGGGGGTTTGGGAATTGATTGGTTCCT
TTAATATTGGGGGCACCTGATATAGCTTTTCCTCGAATAAATAATTTGAGTTTTTGGTTATTACCCCCTT
CATTGTTTATGTTATTTATATCTTCTATAGTAGAAATAGGTGTAGGAACAGGGTGAACTGTATATCCTCC
TTTAGCTTCATTAATAGGTCATGCGGGGAGATCAGTAGATTTCGCTATTTTTTCATTACATTTAGCTGGA
GCTTCTTCTATCATAGGGGCTATTAATTTTATTACAACTGTAATTAATATACGTTCATATGGGATGACTAT
GGAGAAGGTACCGTTATTTGTTTGATCTGTATTAATTACTGCAGTATTATTATTGTTATCTTTACCTGTTTT
AGCAGGTGCTATTACAATATTGTTAACTGATCGAAATTTTAATACATCGTTTTTTGACCCTGCTGGGGG
AGGAGACCCTATTTTATTTCAACATTTATTT

>SAJ286_*E.*sp_ARM

NNNNNNNNNNNNNNNNNNNNNNNNNNNTTGATCAGCTATAGTTGGGACTTCGATAAGAATAATTATTC
GAACGGAATTAGGACAATCTGGGAGATTATTAGGGGATGATCATTTGTATAATGTTGTTGTTACTGCTC
ATGCTTTTGTGATAATTTTTTTTATAGTTATGCCAATTATGATTGGGGGGTTTGGGAATTGATTAGTTCCTT
TAATGTTGGGGGCACCTGATATAGCTTTTCCTCGAATAAATAATTTGAGTTTTTGGTTACTACCCCCTT
CATTGTTTATGTTATTTATATCTTCTATAGTAGAAATGGGTGTCGGAACAGGGTGAACTGTGTATCCTCC
TTTAGCCTCATTGATAGGTCATGCGGGGAGATCAGTAGATTTCGCTATTTTTTCATTACATTTAGCTGG
GGCTTCTTCTATTATAGGGGCTATTAATTTTATTACAACTGTAATTAATATGCGTTCATATGGGATAACTA
TAGAGAAGGTACCGTTATTTGTTTGATCTGTATTGATTACTGCGGTATTATTATTGTTATCTTTACCTGTTT
TAGCAGGTGCTATTACAATATTGTTAACTGATCGAAATTTTAATACATCGTTTTTTGACCCTGCTGGGG
GAGGAGACCCTATTTTATTTCAACATTTATTT

>SAJ298_*E. robin* SAJ298_IRN

NNNNNNNNNNNNNNNNNNNNNNNNTGATCAGCTATAGTTGGGACTTCAATAAGAATAATTATTC
GAACGGAACTAGGACAATCTGGGAGATTATTAGGGGATGATCATTTGTATAATGTTGTTGTTACTGCTC
ATGCTTTTGTTATAATTTTTTTTATAGTTATACCAATTATGATTGGGGGGTTTGGAAACTGATTAGTTCCTT
TAATATTGGGGGCGCCTGATATAGCTTTTCCTCGAATAAATAATTTGAGTTTTTGGTTATTACCTCCTTC
ATTGTTTATGTTATTTATATCTTCTATAGTAGAAATGGGTGTAGGAACAGGGTGAACTGTATATCCTCCT
TTAGCTTCATTAATAGGTCATGCGGGGAGATCAGTAGATTTTGCTATTTTTTCATTACATTTAGCTGGGG
CTTCTTCTATTATAGGGGCTATTAATTTTATTACAACTGTAATTAATATACGTTCATATGGGATGACTATA
GAGAAGGTACCGTTATTTGTTTGATCTGTATTGATTACTGCGGTGTTATTATTATTATCTTTACCTGTTTTA
GCAGGTGCTATTACAATATTATTAACTGATCGAAATTTTAATACATCGTTTTTTGACCCTGCTGGGGGA
GGAGATCCTATTTTATTTCAACATTTATTT

>SAJ300_*E.*_*sparabara*_IRN

NNNNNNNNNNNNNNNNNNNGGAGCTTGATCAGCTATAGTTGGAACTTCAATAAGAATGATTATTC
GAACGGAATTAGGACAGTCTGGGAGATTATTAGGGGATGATCATTTGTATAATGTTGTTGTTACTGCTC
ATGCTTTTGTGATAATTTTTTTTATAGTTATACCAATTATGATTGGGGGGTTTGGGAATTGATTAGTTCCTT
TAATATTGGGGGCACCTGATATAGCTTTTCCTCGGATAAATAATTTGAGTTTTTGGTTATTACCCCCTTC
ATTATTTATATTATTTATATCTTCTATAGTAGAAATGGGTGTAGGAACAGGGTGAACTGTTTATCCTCCTT
TAGCCTCATTAATAGGTCATGCGGGGGGATCAGTAGATTTCGCTATTTTTTCATTACATTTAGCTGGGG
CTTCTTCTATTATAGGGGCTATTAATTTTATTACAACTGTAATTAATATACGTTCATATGGGATGACTATA
GAGAAGGTACCGTTATTTGTTTGATCTGTATTAATTACTGCGGTATTGTTATTGTTATCTTTACCTGTTTTA
GCGGGTGCTATTACAATGTTGTTAACTGATCGAAATTTTAATACATCGTTTTTTGATCCTGCTGGGGGA
GGAGATCCTATTTTATTTCAACATTTATTT

>SAJ319_*E.marmoratus*_IRN

NNNNNNNNNNNNNNNNNNGGGAGCTTGATCGGCTATAGTTGGAACTTCAATAAGAATAATTATTC
GAACAGAATTAGGACAGTCTGGAAGATTATTAGGAGATGATCATCTATATAATGTTGTTGTTACTGCTC
ATGCTTTTGTAATGATTTTTTTTATAGTTATACCAATTTTAATTGGTGGGTTTGGAAATTGGTTGGTCCCAT
TAATATTAGGAGCACCTGATATAGCTTTTCCTCGAATAAATAATTTAAGTTTTTGGTTGTTACCTCCTTCT
TTGTTTATATTATTTATATCTTCTATAGTAGAAATAGGTGTGGGGACAGGATGAACTGTGTATCCTCCTTT
AGCTTCATTAATAGGTCATGCGGGGAGATCAGTAGATTTTGCTATTTTTTCATTACATTTAGCTGGGGC
TTCTTCTATTATAGGGGCTATTAATTTTATTACAACTGTAATTAATATACGTTCATATGGAATAACTATGGA
GAAGGTACCGTTATTTGTTTGATCTGTATTAATTACTGCAGTGTTATTATTATTATCTTTACCTGTTTTGGC
AGGTGCTATTACTATATTATTAACTGATCGAAATTTTAATACGTCGTTTTTTGATCCTGCTGGAGGGGGG
GATCCTATTTTATTTCAACACTTATTT

>SAJ320_E.rezaci._IRN

NNNNNNNNNNNNNNNNNNNNNNGCTTGATCAGCTATAGTTGGAACTTCAATAAGAATGATTATTC
GAACGGAATTAGGACAATCTGGGAGATTATTAGGAGATGATCATTTATATAATGTTGTTGTTACTGCTC
ATGCTTTTGTGATGATTTTTTTTATGGTTATACCAATTATAATTGGGGGATTTGGGAATTGGTTGGTTCCTT
TAATATTAGGAGCACCTGATATAGCTTTCCCTCGAATAAATAATTTGAGTTTTTGGTTATTACCTCCTTCT
TTATTTATACTATTTATATCTTCTATGGTAGAAATGGGTGTAGGAACAGGGTGAACTGTTTATCCCCCTT
TAGCCTTATTGATAGGTCATGCGGGGAGATCAGTAGATTTTGCTATTTTTTCGTTACATTTAGCTGGGG
CTTCTTCTATTATAGGAGCTATTAATTTTATTACAACTGTAATTAATATGCGTTCATATGGAATGACTATAG
AGAAGGTGCCGTTGTTTGTTTGATCTGTATTAATTACTGCGGTATTATTATTATTATCTTTACCTGTTTTAG
CAGGTGCTATTACGATATTATTAACTGATCGAAATTTTAATACATCGTTTTTTGATCCTGCTGGGGGAG
GAGATCCTATTTTATTTCAACATTTATTT

>SAJ321_*E.athanatoi*._IRN

NNNNNNNNNNNNNNNNNNNNNNNAGCTTGATCAGCTATGGTTGGGACTTCAATAAGAATAATTATTC
GAACGGAATTAGGGCAATCTGGGAGATTATTAGGGGATGATCATTTGTATAATGTTGTTGTTACTGCTC
ATGCTTTTGTAATAATTTTTTTTATAGTTATACCAATTATAATTGGAGGGTTTGGGAATTGACTGGTTCCTT
TAATGTTAGGAGCACCTGATATAGCTTTTCCTCGAATAAATAATTTGAGTTTTTGGTTATTGCCCCCTTC
ATTATTTATGTTATTTATATCTTCTATAGTGGAAATAGGTGTAGGAACAGGATGAACTGTTTATCCCCCTT
TAGCCTCGTTAATAGGTCATGCGGGGAGATCAGTAGATTTTGCTATTTTTTCATTACATTTAGCTGGGG
CTTCTTCTATTATAGGAGCTATTAATTTCATTACAACTGTGATTAATATGCGTTCATATGGAATAAGTATA
GAGAAGGTACCGTTATTTGTTTGATCTGTATTAATTACTGCGGTATTGTTGTTGTTATCATTACCTGTTTT
AGCGGGTGCTATTACGATATTGTTAACTGATCGAAATTTTAATACATCGTTTTTTGATCCTGCTGGGGG
AGGAGATCCTATTTTATTTCAACATTTATTT

>SAJ366_*E.transcaucasicus*_ARM

NNNNNNNNNNNNNNNNNNNNNNNNNNNNNNNNCAGCTATAGTTGGGACTTCAATAAGAATAATTATT
CGGACGGAATTAGGACAATCTGGGAGATTATTGGGGGATGATCATTTGTATAATGTTGTTGTTACTGCT
CATGCTTTTGTGATAATTTTTTTTATAGTTATACCAATTATGATTGGGGGGTTTGGGAATTGATTAGTTCCT
TTAATGTTGGGGGCGCCTGATATAGCTTTTCCTCGAATAAATAATTTGAGTTTTTGGTTATTACCCCCTT
CATTATTTATGTTATTTATATCTTCTATAGTAGAAATGGGTGTAGGAACAGGGTGAACTGTGTATCCTCC
TTTAGCCTCATTAATAGGTCATGCGGGGAGATCAGTAGATTTCGCTATTTTTTCATTACATTTAGCTGG
GGCTTCTTCTATTATAGGGGCTATTAATTTTATTACAACTGTAATTAATATACGTTCATATGGGATGACTA
TAGAGAAGGTGCCGTTATTTGTTTGATCTGTATTAATTACTGCGGTGTTATTGTTGTTATCTTTACCTGTT
TTAGCGGGTGCTATTACAATATTGTTAACTGATCGAAATTTTAATACATCGTTTTTTGACCCTGCTGGGG
GAGGAGATCCTATTTTATTTCAACATTTATTT

>SAJ367_*E.transcaucasicus*_ARM

NNNNNNNNNNNNNNNNNNNNNNNAGCTTGATCAGCTATAGTTGGGACTTCAATAAGAATAATTATTC
GGACGGAATTAGGACAATCTGGGAGATTATTGGGGGATGATCATTTGTATAATGTTGTTGTTACTGCTC
ATGCTTTTGTGATAATTTTTTTTATAGTTATACCAATTATGATTGGGGGGTTTGGGAATTGATTAGTTCCTT
TAATGTTGGGGGCGCCTGATATAGCTTTTCCTCGAATAAATAATTTGAGTTTTTGGTTATTACCCCCTTC
ATTATTTATGTTATTTATATCTTCTATAGTAGAAATGGGTGTAGGAACAGGGTGAACTGTGTATCCTCCT
TTAGCCTCATTAATAGGTCATGCGGGGAGATCAGTAGATTTCGCTATTTTTTCATTACATTTAGCTGGG
GCTTCTTCTATTATAGGGGCTATTAATTTTATTACAACTGTAATTAATATACGTTCATATGGGATGACTAT
AGAGAAGGTGCCGTTATTTGTTTGATCTGTATTAATTACTGCGGTGTTATTGTTGTTATCTTTACCTGTTT
TAGCGGGTGCTATTACAATATTATTAACTGATCGAAATTTTAATACATCGTTTTTTGACCCTGCTGGGG
GAGGAGATCCTATTTTATTTCAACATTTATTT

>SMNKARA08341_*E.kollari*_GER

NNCATTATATTTAATTTTTGGAGCTTGGTCGGCTATGGTTGGAACTTCAATAAGAATAATTATTCGAACA
GAATTAGGACAATCTGGGAGATTATTAGGAGATGATCATCTGTATAATGTTGTTGTTACTGCTCATGCTT
TTGTAATGATTTTTTTTATAGTTATACCAATTTTAATTGGAGGGTTTGGGAATTGATTAGTTCCTTTAATATT
AGGAGCACCTGATATAGCTTTTCCTCGAATAAATAATTTAAGTTTTTGGTTGTTACCTCCTTCATTGTTTA
TATTATTTATGTCTTCTATGGTAGAGATGGGTGTAGGAACAGGGTGAACTGTTTATCCTCCTTTGGCTT
CATTAATAGGCCATTCGGGAAGATCAGTGGATTTTGCTATTTTTTCATTACATTTAGCTGGGGCTTCTTC
TATTATAGGAGCTATTAATTTCATTACAACTGTAATTAATATACGTTCATATGGAATAACTATAGAGAAGG
TACCATTATTTGTTTGGTCTGTATTAATTACTGCAGTATTATTATTACTATCTTTACCTGTTTTAGCAGGTG
CTATTACAATATTATTAACTGATCGAAATTTTAATACATCGTTTTTTGACCCTGCTGGGGGAGGGGACC
CGATTTTATTTCAACATTTATTT

>SPICR045-10_JF886220_*E.kollari*_RUS

NNCATTGTATTTAATTTTTGGAGCTTGATCAGCTATAGTTGGAACTTCAATAAGAATAATTATTCGAACG
GAATTAGGACAATCTGGGAGATTATTAGGGGATGATCATTTGTATAATGTTGTTGTTACTGCTCATGCTT
TTGTGATAATTTTTTTTATAGTTATACCAATTATAATTGGGGGGTTTGGAAATTGATTGGTTCCTTTAATGTT
AGGGGCACCTGATATAGCTTTTCCTCGAATAAATAATTTGAGTTTTTGGTTATTACCTCCTTCATTGTTTA
TATTATTTATGTCTTCTATAGTAGAAATAGGTGTAGGGACAGGGTGAACTGTTTATCCTCCTCTAGCGT
CATTAATAGGTCATGCAGGGAGATCAGTAGATTTTGCTATTTTTTCATTACATTTAGCTGGGGCTTCTTC
TATTATGGGGGCTATTAATTTTATTACAACTGTAATTAATATACGTTCATATGGAATAACAATGGAGAAG
GTATCATTATTTGTTTGATCTGTTTTAATTACTGCGGTATTATTATTGTTATCTTTACCTGTTTTAGCGGGT
GCTATTACAATATTGTTAACTGATCGAAATTTTAATACATCGTTTTTTGACCCTGCTGGGGGAGGAGAT
CCTATTTTATTTCAACATTTATTT

Commandline for IQTREE: iqtree2 -s ERETG2.fas -m TESTNEW -bb 1000 -alrt 1000

Resulted tree:

(AR451-
10_HQ982805_BATMAN_TUR:0.0063866165,(((((((BJ001_E.granosus:0.0194459471,((((KX53708
3_E.cinnaberinus_GER:0.0019700810,OL352232.1_E.kollari_SAJ26:0.0026577484)81:0.001555
4310,OL352226.1_E.kollari_SAJ128_CHI:0.0052573421)99:0.0103076916,SPICR045-
10_JF886220_E.kollari_RUS:0.0093493352)94:0.0048911039,(KY268507.1_E.moravicus_AUS:0.
0000024859,OL352218.1_E.moravicus_SAJ099:0.0000024859)100:0.0393747751)80:0.009188
9157)71:0.0063482815,((((((DQ973153_E.cinnaberinus_Ecinwest1_GER:0.0000024892,(OL3522
35.1_E.cf._kollari_SAJ38:0.0000024859,SMNKARA08341_E.kollari_GER:0.0000024859)92:0.005
1101984)99:0.0364351691,(OL352224.1_E.lavrosiae_SAJ12:0.0367597168,OP434393.1_E.yuku
ni_YJL-
2022b_20220914_CHI:0.0528969593)70:0.0050645804)78:0.0141746126,((FJ948997.1_E.sp._1
3_06_ISR:0.1274242647,(FJ948999.1_E.walckenaeri_14_5_GRE:0.0200010893,OL352229.1_E.
walckenaeri_SAJ14_TUR:0.0157103995)100:0.0783533364)96:0.0528761338,(FJ948998.1_E.cf.
_kollari_14_04_ITA:0.0430679026,OL352228.1_E.sp._SAJ130_ITA:0.0373382002)90:0.01386515
28)51:0.0104326331)18:0.0000023336,SAJ106_E.agrinus_IRN:0.0811374734)40:0.0070773544,
(OP376824.1_E.da_20220728_CHI:0.0119766903,SAJ319_E.sp._IRN:0.0000021192)100:0.0710
942694)83:0.0113228219,SAJ320_E.sp._IRN:0.0449776354)62:0.0055731431)73:0.0080759726
,SAJ321_E.sp._IRN:0.0475780167)84:0.0121427787,SAJ300_E.sp_BW_IRN:0.0142484222)65:0.
0054123388,SAJ088_E.surena_IRN:0.0071386305)64:0.0025875280,((KX443582.1_E.sandaliatu
s_DK:0.0000024859,((OL352220.1_E.hermani_SAJ103:0.0031164312,SAJ175_E.hermani:0.0032
013734)99:0.0032321123,OL352231.1_E.sandaliatus_SAJ17_NED:0.0133523098)81:0.0028280
743)98:0.0062975995,(OL352230.1_E.algarvensis_SAJ15:0.0014894535,SAJ286_E.sp_ARM:0.00
31188455)100:0.0080805536)94:0.0032386815)65:0.0029226931,(((CaBOL_1018700:0.001462
0894,CaBOL_1018701:0.0014618196)39:0.0000024859,SAJ366_E.sp_ARM:0.0000024859)41:0.
0000024859,SAJ367_E.sp_ARM:0.0015244739)97:0.0095973838)97:0.0152331038,SAJ298_E.s
p_IRN:0.0054603133);

Detailed result of the analysis:

IQ-TREE 3.0.1 built May 5 2025

Input file name: ERETG2.fas

Type of analysis: ModelFinder + tree reconstruction + ultrafast bootstrap (1000 replicates)

Random seed number: 874532

REFERENCES

To cite IQ-TREE 3 please use:

Thomas K.F. Wong, Nhan Ly-Trong, Huaiyan Ren, Hector Banos, Andrew J. Roger,
Edward Susko, Chris Bielow, Nicola De Maio, Nick Goldman, Matthew W. Hahn,
Gavin Huttley, Robert Lanfear, Bui Quang Minh (2025)
IQ-TREE 3: Phylogenomic Inference Software using Complex Evolutionary Models.
Submitted.

Please also cite the following paper(s) for the feature(s) that you used:

To cite ModelFinder please use:

Subha Kalyaanamoorthy, Bui Quang Minh, Thomas KF Wong, Arndt von Haeseler,
and Lars S Jermiin (2017) ModelFinder: Fast model selection for
accurate phylogenetic estimates. Nature Methods, 14:587–589.
https://doi.org/10.1038/nmeth.4285

Since you used ultrafast bootstrap (UFBoot) please also cite:

Diep Thi Hoang, Olga Chernomor, Arndt von Haeseler, Bui Quang Minh,
and Le Sy Vinh (2018) UFBoot2: Improving the ultrafast bootstrap

approximation. Mol. Biol. Evol., 35:518–522.

https://doi.org/10.1093/molbev/msx281

SEQUENCE ALIGNMENT

Input data: 36 sequences with 658 nucleotide sites

Number of constant sites: 483 (= 73.4043% of all sites)

Number of invariant (constant or ambiguous constant) sites: 483 (= 73.4043% of all sites)

Number of parsimony informative sites: 128

Number of distinct site patterns: 208

ModelFinder

Best-fit model according to BIC: TN+F+I+G4

List of models sorted by BIC scores:

Model	LogL	AIC		w-AIC	AICc		w-AICc	BIC		w-BIC
TN+F+I+G4	-2988.324	6128.648	+	0.101	6148.793	+	0.139	6469.828	+	0.557
TIM+F+I+G4	-2985.916	6125.832	+	0.412	6146.543	+	0.429	6471.501	+	0.241
TIM2+F+I+G4	-2986.779	6127.559	+	0.174	6148.269	+	0.181	6473.227	+	0.102
TIM3+F+I+G4	-2987.157	6128.313	+	0.119	6149.024	+	0.124	6473.982	+	0.0698
TN+F+R2	-2991.862	6135.725	-	0.00293	6155.869	-	0.00405	6476.904	-	0.0162
TIM+F+R2	-2989.635	6133.270	-	0.00999	6153.980	-	0.0104	6478.939	-	0.00586
TIM3+F+R2	-2989.888	6133.775	-	0.00776	6154.486	-	0.00809	6479.444	-	0.00455
TIM2+F+R2	-2990.558	6135.116	-	0.00397	6155.826	-	0.00414	6480.784	-	0.00233
GTR+F+I+G4	-2984.825	6127.650	+	0.166	6149.518	+	0.097	6482.297	-	0.00109
GTR+F+R2	-2988.545	6135.090	-	0.00402	6156.959	-	0.00235	6489.738	-	2.65e-05
HKY+F+I+G4	-3011.073	6172.147	-	3.61e-11	6191.734	-	6.6e-11	6508.837	-	1.88e-09

K3Pu+F+I+G4 -3008.961 6169.921 - 1.1e-10 6190.066 - 1.52e-10 6511.101 - 6.08e-10

TPM2u+F+I+G4 -3009.799 6171.598 - 4.75e-11 6191.743 - 6.57e-11 6512.778 - 2.63e-10

TPM3u+F+I+G4 -3009.983 6171.967 - 3.95e-11 6192.112 - 5.47e-11 6513.146 - 2.18e-10

HKY+F+R2 -3016.237 6182.473 - 2.07e-13 6202.061 - 3.78e-13 6519.164 - 1.08e-11

K3Pu+F+R2 -3014.948 6181.896 - 2.76e-13 6202.041 - 3.82e-13 6523.076 - 1.52e-12

TVM+F+I+G4 -3008.545 6173.091 - 2.25e-11 6194.376 - 1.76e-11 6523.249 - 1.4e-12

TPM3u+F+R2 -3015.744 6183.488 - 1.24e-13 6203.633 - 1.72e-13 6524.668 - 6.88e-13

TPM2u+F+R2 -3015.817 6183.634 - 1.16e-13 6203.779 - 1.6e-13 6524.814 - 6.4e-13

TVM+F+R2 -3013.948 6183.896 - 1.01e-13 6205.181 - 7.94e-14 6534.054 - 6.3e-15

TIM3+I+G4 -3057.466 6262.933 - 6.98e-31 6281.972 - 1.68e-30 6595.134 - 3.44e-28

TIM3e+I+G4 -3057.541 6263.081 - 6.48e-31 6282.121 - 1.56e-30 6595.282 - 3.19e-28

TIM3e+I+G4 -3057.541 6263.081 - 6.48e-31 6282.121 - 1.56e-30 6595.282 - 3.19e-28

GTR+I+G4 -3053.224 6258.449 - 6.57e-30 6278.593 - 9.09e-30 6599.628 - 3.63e-29

SYM+I+G4 -3053.447 6258.895 - 5.25e-30 6279.039 - 7.27e-30 6600.074 - 2.91e-29

SYM+I+G4 -3053.447 6258.895 - 5.25e-30 6279.039 - 7.27e-30 6600.074 - 2.91e-29

TIM+I+G4 -3060.485 6268.969 - 3.41e-32 6288.008 - 8.2e-32 6601.170 - 1.68e-29

TIMe+I+G4 -3060.660 6269.321 - 2.86e-32 6288.360 - 6.88e-32 6601.522 - 1.41e-29

TIMe+I+G4 -3060.660 6269.321 - 2.86e-32 6288.360 - 6.88e-32 6601.522 - 1.41e-29

TIM3+R2 -3062.250 6272.501 - 5.83e-33 6291.540 - 1.4e-32 6604.702 - 2.87e-30

TIM3e+R2 -3062.265 6272.531 - 5.75e-33 6291.570 - 1.38e-32 6604.732 - 2.83e-30

TIM3e+R2 -3062.265 6272.531 - 5.75e-33 6291.570 - 1.38e-32 6604.732 - 2.83e-30

TN+I+G4 -3068.592 6283.185 - 2.79e-35 6301.685 - 8.79e-35 6610.897 - 1.3e-31

TIM2+I+G4 -3065.400 6278.800 - 2.5e-34 6297.840 - 6.01e-34 6611.002 - 1.23e-31

TNe+I+G4 -3068.657 6283.314 - 2.62e-35 6301.814 - 8.24e-35 6611.026 - 1.22e-31

TNe+I+G4 -3068.657 6283.314 - 2.62e-35 6301.814 - 8.24e-35 6611.026 - 1.22e-31

TIM2e+I+G4 -3065.597 6279.194 - 2.05e-34 6298.234 - 4.94e-34 6611.395 - 1.01e-31

TIM2e+I+G4 -3065.597 6279.194 - 2.05e-34 6298.234 - 4.94e-34 6611.395 - 1.01e-31

GTR+R2 -3059.135 6270.269 - 1.78e-32 6290.414 - 2.46e-32 6611.449 - 9.85e-32

SYM+R2 -3059.149 6270.297 - 1.76e-32 6290.442 - 2.43e-32 6611.477 - 9.71e-32

SYM+R2 -3059.149 6270.297 - 1.76e-32 6290.442 - 2.43e-32 6611.477 - 9.71e-32

TIM+R2 -3067.419 6282.839 - 3.32e-35 6301.878 - 7.98e-35 6615.040 - 1.63e-32

TIMe+R2	-3067.485	6282.971 - 3.11e-35	6302.010 - 7.47e-35	6615.172 - 1.53e-32
TIMe+R2	-3067.485	6282.971 - 3.11e-35	6302.010 - 7.47e-35	6615.172 - 1.53e-32
TN+R2	-3073.297	6292.593 - 2.53e-37	6311.093 - 7.96e-37	6620.305 - 1.18e-33
TNe+R2	-3073.359	6292.717 - 2.38e-37	6311.217 - 7.48e-37	6620.429 - 1.1e-33
TNe+R2	-3073.359	6292.717 - 2.38e-37	6311.217 - 7.48e-37	6620.429 - 1.1e-33
TIM2+R2	-3071.298	6290.596 - 6.86e-37	6309.636 - 1.65e-36	6622.798 - 3.38e-34
TIM2e+R2	-3071.319	6290.637 - 6.73e-37	6309.677 - 1.62e-36	6622.838 - 3.31e-34
TIM2e+R2	-3071.319	6290.637 - 6.73e-37	6309.677 - 1.62e-36	6622.838 - 3.31e-34
TPM3u+I+G4	-3099.626	6345.253 - 9.29e-49	6363.753 - 2.93e-48	6672.965 - 4.32e-45
TPM3+I+G4	-3099.811	6345.622 - 7.73e-49	6364.122 - 2.43e-48	6673.334 - 3.59e-45
TPM3+I+G4	-3099.811	6345.622 - 7.73e-49	6364.122 - 2.43e-48	6673.334 - 3.59e-45
TVM+I+G4	-3098.314	6346.628 - 4.67e-49	6366.216 - 8.54e-49	6683.318 - 2.44e-47
TVMe+I+G4	-3098.435	6346.870 - 4.14e-49	6366.458 - 7.56e-49	6683.561 - 2.16e-47
TVMe+I+G4	-3098.435	6346.870 - 4.14e-49	6366.458 - 7.56e-49	6683.561 - 2.16e-47
TPM3+R2	-3106.233	6358.466 - 1.26e-51	6376.966 - 3.95e-51	6686.178 - 5.84e-48
TPM3+R2	-3106.233	6358.466 - 1.26e-51	6376.966 - 3.95e-51	6686.178 - 5.84e-48
TPM3u+R2	-3106.234	6358.467 - 1.26e-51	6376.967 - 3.95e-51	6686.179 - 5.83e-48
K3Pu+I+G4	-3107.669	6361.338 - 2.99e-52	6379.838 - 9.41e-52	6689.050 - 1.39e-48
K3P+I+G4	-3107.869	6361.739 - 2.44e-52	6380.239 - 7.7e-52	6689.451 - 1.14e-48
K3P+I+G4	-3107.869	6361.739 - 2.44e-52	6380.239 - 7.7e-52	6689.451 - 1.14e-48
HKY+I+G4	-3113.632	6371.265 - 2.09e-54	6389.234 - 8.57e-54	6694.487 - 9.16e-50
K2P+I+G4	-3113.633	6371.265 - 2.09e-54	6389.234 - 8.57e-54	6694.488 - 9.15e-50
K2P+I+G4	-3113.633	6371.265 - 2.09e-54	6389.234 - 8.57e-54	6694.488 - 9.15e-50
TVM+R2	-3104.551	6359.102 - 9.14e-52	6378.689 - 1.67e-51	6695.792 - 4.77e-50
TVMe+R2	-3104.554	6359.108 - 9.11e-52	6378.695 - 1.67e-51	6695.798 - 4.75e-50
TVMe+R2	-3104.554	6359.108 - 9.11e-52	6378.695 - 1.67e-51	6695.798 - 4.75e-50
TPM2u+I+G4	-3112.229	6370.458 - 3.12e-54	6388.958 - 9.84e-54	6698.170 - 1.45e-50
TPM2+I+G4	-3112.272	6370.543 - 2.99e-54	6389.043 - 9.43e-54	6698.255 - 1.39e-50
TPM2+I+G4	-3112.272	6370.543 - 2.99e-54	6389.043 - 9.43e-54	6698.255 - 1.39e-50
K3Pu+R2	-3114.118	6374.237 - 4.72e-55	6392.737 - 1.49e-54	6701.949 - 2.2e-51
K3P+R2	-3114.250	6374.501 - 4.14e-55	6393.001 - 1.3e-54	6702.213 - 1.92e-51

K3P+R2 -3114.250 6374.501 - 4.14e-55 6393.001 - 1.3e-54 6702.213 - 1.92e-51

HKY+R2 -3119.759 6383.518 - 4.56e-57 6401.487 - 1.87e-56 6706.740 - 2e-52

K2P+R2 -3119.759 6383.519 - 4.56e-57 6401.488 - 1.87e-56 6706.741 - 2e-52

K2P+R2 -3119.759 6383.519 - 4.56e-57 6401.488 - 1.87e-56 6706.741 - 2e-52

TPM2u+R2 -3118.761 6383.522 - 4.55e-57 6402.022 - 1.43e-56 6711.234 - 2.11e-53

TPM2+R2 -3118.907 6383.815 - 3.93e-57 6402.315 - 1.24e-56 6711.527 - 1.83e-53

TPM2+R2 -3118.907 6383.815 - 3.93e-57 6402.315 - 1.24e-56 6711.527 - 1.83e-53

F81+F+I+G4 -3387.331 6922.663 - 3.85e-174 6941.702 - 9.25e-174 7254.864 - 1.89e-171

F81+F+R2 -3390.538 6929.077 - 1.56e-175 6948.116 - 3.74e-175 7261.278 - 7.67e-173

F81+I+G4 -3410.692 6963.383 - 5.53e-183 6980.830 - 2.95e-182 7282.117 - 2.29e-177

JC+I+G4 -3410.714 6963.428 - 5.41e-183 6980.875 - 2.88e-182 7282.162 - 2.24e-177

JC+I+G4 -3410.714 6963.428 - 5.41e-183 6980.875 - 2.88e-182 7282.162 - 2.24e-177

JC+R2 -3414.679 6971.359 - 1.03e-184 6988.806 - 5.47e-184 7290.092 - 4.24e-179

JC+R2 -3414.679 6971.359 - 1.03e-184 6988.806 - 5.47e-184 7290.092 - 4.24e-179

F81+R2 -3414.681 6971.362 - 1.02e-184 6988.809 - 5.46e-184 7290.095 - 4.24e-179

JC+I+R2 -3413.190 6970.381 - 1.67e-184 6988.350 - 6.87e-184 7293.603 - 7.33e-180

JC+G4 -3419.914 6979.828 - 1.49e-186 6996.762 - 1.02e-185 7294.072 - 5.8e-180

JC+I+R3 -3409.686 6967.372 - 7.53e-184 6986.411 - 1.81e-183 7299.573 - 3.71e-181

JC+R3 -3414.399 6974.798 - 1.84e-185 6993.298 - 5.78e-185 7302.510 - 8.54e-182

JC+I -3436.486 7012.973 - 9.43e-194 7029.906 - 6.5e-193 7327.217 - 3.68e-187

JC -3719.675 7577.350 - 2.64e-316 7593.779 - 2.34e-315 7887.105 - 9.73e-309

AIC, w-AIC : Akaike information criterion scores and weights.

AICc, w-AICc : Corrected AIC scores and weights.

BIC, w-BIC : Bayesian information criterion scores and weights.

Plus signs denote the 95% confidence sets.

Minus signs denote significant exclusion.

SUBSTITUTION PROCESS

Model of substitution: TN+F+I+G4

Rate parameter R:

A-C: 1.0000

A-G: 32.8210

A-T: 1.0000

C-G: 1.0000

C-T: 7.7279

G-T: 1.0000

State frequencies: (empirical counts from alignment)

pi(A) = 0.2579

pi(C) = 0.1277

pi(G) = 0.2001

pi(T) = 0.4142

Rate matrix Q:

A	-1.512	0.02717	1.397	0.0881
C	0.05484	-0.7782	0.04256	0.6808
G	1.8	0.02717	-1.915	0.0881
T	0.05484	0.2099	0.04256	-0.3074

Model of rate heterogeneity: Invar+Gamma with 4 categories

Proportion of invariable sites: 0.6056

Gamma shape alpha: 1.352

Category Relative_rate Proportion

0 0 0.6056

1 0.5108 0.09859

2 1.425 0.09859

3 2.638 0.09859

4 5.57 0.09859

Relative rates are computed as MEAN of the portion of the Gamma distribution falling in the category.

MAXIMUM LIKELIHOOD TREE

Log-likelihood of the tree: -2983.1559 (s.e. 145.7985)

Unconstrained log-likelihood (without tree): -2618.0157

Number of free parameters (#branches + #model parameters): 76

Akaike information criterion (AIC) score: 6118.3117

Corrected Akaike information criterion (AICc) score: 6138.4563

Bayesian information criterion (BIC) score: 6459.4913

Total tree length (sum of branch lengths): 1.0842

Sum of internal branch lengths: 0.4189 (38.6386% of tree length)

WARNING: 3 near-zero internal branches (<0.0015) should be treated with caution

Such branches are denoted by '**' in the figure below

NOTE: Tree is UNROOTED although outgroup taxon 'AR451-10_HQ982805_BATMAN_TUR' is drawn at root

Numbers in parentheses are SH-aLRT support (%) / ultrafast bootstrap support (%)

```
+--AR451-10_HQ982805_BATMAN_TUR
|
|          +---BJ001_E.granosus
|        +--| (56.3/71)
```

```
|           | |       +--KX537083_E.cinnaberinus_GER
|           | |     +--| (37.6/81)
|           | |     | +--OL352232.1_E.kollari_SAJ26
|           | |   +--| (92.6/99)
|           | |   | +--OL352226.1_E.kollari_SAJ128_CHI
|           | | +--| (79.9/94)
|           | | | +--SPICR045-10_JF886220_E.kollari_RUS
|           | +--| (69.1/80)
|           |   |     +**KY268507.1_E.moravicus_AUS
|           |   +--------| (100/100)
|           |         +**OL352218.1_E.moravicus_SAJ099
|         +--| (75.9/73)
|         | |             +**DQ973153_E.cinnaberinus_Ecinwest1_GER
|         | |         +-------| (98.7/99)
|         | |         |     | +**OL352235.1_E.cf._kollari_SAJ38
|         | |         |     +--| (34.2/92)
|         | |         |       +**SMNKARA08341_E.kollari_GER
|         | |       +--| (84.4/78)
|         | |       | | +-------OL352224.1_E.lavrosiae_SAJ12
|         | |       | +--| (38.1/70)
|         | |       |   +-----------OP434393.1_E.yukuni_YJL-2022b_20220914_CHI
|         | |     +**| (0/18)
|         | |     | |         +---------------------------FJ948997.1_E.sp._13_06_ISR
|         | |     | | +-----------| (92.4/96)
|         | |     | | |       |           +---FJ948999.1_E.walckenaeri_14_5_GRE
|         | |     | | |       +-----------------| (98.4/100)
|         | |     | | |                   +--OL352229.1_E.walckenaeri_SAJ14_TUR
|         | |     | +--| (14.3/51)
|         | |     |   | +--------FJ948998.1_E.cf._kollari_14_04_ITA
|         | |     |   +--| (79.5/90)
|         | |     |     +-------OL352228.1_E.sp._SAJ130_ITA
```

```
|          | |    +--| (79.2/40)
|          | |    | +-----------------SAJ106_E.agrinus_IRN
|          | | +--| (57.6/83)
|          | | | |          +--OP376824.1_E.da_20220728_CHI
|          | | | +---------------| (100/100)
|          | | |            +**SAJ319_E.sp._IRN
|          | +--| (70.6/62)
|          |    +---------SAJ320_E.sp._IRN
|        +--| (88.1/84)
|        | +---------SAJ321_E.sp._IRN
|      +--| (80.9/65)
|      | +--SAJ300_E.sp_BW_IRN
|    +--| (78.2/64)
|    | +--SAJ088_E.surena_IRN
| +--| (79.2/65)
| | |    +**KX443582.1_E.sandaliatus_DK
| | | +--| (95/98)
| | | | | |    +--OL352220.1_E.hermani_SAJ103
| | | | | | +--| (81/99)
| | | | | | | +--SAJ175_E.hermani
| | | | +--| (35.6/81)
| | | |    +--OL352231.1_E.sandaliatus_SAJ17_NED
| | +--| (83.9/94)
| |    | +**OL352230.1_E.algarvensis_SAJ15
| |    +--| (94.4/100)
| |      +--SAJ286_E.sp_ARM
+--| (98.8/97)
| |    +**CaBOL_1018700
| | +**| (0/36)
| | | | +**SAJ366_E.sp_ARM
| | | +**| (0/40)
```

| | | +--SAJ367_E.sp_ARM

| +--| (96.5/97)

| +**CaBOL_1018701

|

+--SAJ298_E.sp_IRN

Tree in newick format:

(AR451-10_HQ982805_BATMAN_TUR:0.0063919279,(((((((BJ001_E.granosus:0.0194661776,((((KX537083_E.cinnaberinus_GER:0.0019716021,OL352232.1_E.kollari_SAJ26:0.0026617922)37.6/81:0.0015573625,OL352226.1_E.kollari_SAJ128_CHI:0.0052641066)92.6/99:0.0103194760,SPICR045-10_JF886220_E.kollari_RUS:0.0093600299)79.9/94:0.0048985767,(KY268507.1_E.moravicus_AUS:0.0000010149,OL352218.1_E.moravicus_SAJ099:0.0000010149)100/100:0.0394299444)69.1/80:0.0092029812)56.3/71:0.0063535353,((((((DQ973153_E.cinnaberinus_Ecinwest1_GER:0.0000010149,(OL352235.1_E.cf._kollari_SAJ38:0.0000010149,SMNKARA08341_E.kollari_GER:0.0000010149)34.2/92:0.0051197192)98.7/99:0.0365258602,(OL352224.1_E.lavrosiae_SAJ12:0.0368363288,OP434393.1_E.yukuni_YJL-2022b_20220914_CHI:0.0530295750)38.1/70:0.0050764898)84.4/78:0.0142041528,((FJ948997.1_E.sp._13_06_ISR:0.1277154986,(FJ948999.1_E.walckenaeri_14_5_GRE:0.0201979013,OL352229.1_E.walckenaeri_SAJ14_TUR:0.0156257411)98.4/100:0.0785515836)92.4/96:0.0529744231,(FJ948998.1_E.cf._kollari_14_04_ITA:0.0431740685,OL352228.1_E.sp._SAJ130_ITA:0.0374228233)79.5/90:0.0138637654)14.3/51:0.0104553430)0/18:0.0000021570,SAJ106_E.agrinus_IRN:0.0813011532)79.2/40:0.0070843788,(OP376824.1_E.da_20220728_CHI:0.0119958545,SAJ319_E.sp._IRN:0.0000025261)100/100:0.0712344150)57.6/83:0.0113271652,SAJ320_E.sp._IRN:0.0450458012)70.6/62:0.0055755551)75.9/73:0.0080665675,SAJ321_E.sp._IRN:0.0476421801)88.1/84:0.0121624615,SAJ300_E.sp_BW_IRN:0.0142580379)80.9/65:0.0054202376,SAJ088_E.surena_IRN:0.0071453262)78.2/64:0.0025897403,((KX443582.1_E.sandaliatus_DK:0.0000010149,((OL352220.1_E.hermani_SAJ103:0.0031200009,SAJ175_E.hermani:0.0032051459)81/99:0.0032362241,OL352231.1_E.sandaliatus_SAJ17_NED:0.0133665285)35.6/81:0.0028286613)95/98:0.0063043101,(OL352230.1_E.algarvensis_SAJ15:0.0014910354,SAJ286_E.sp_ARM:0.0031227792)94.4/100:0.0080893668)83.9/94:0.0032413937)79.2/65:0.0029222550,((CaBOL_1018700:0.0014635482,(SAJ366_E.sp_ARM:0.0000010149,SAJ367_E.sp_ARM:0.0015264477)0/40:0.0000010149)0/36:0.0000010149,CaBOL_1018701:0.0014632303)96.5/97:0.0096067865)98.8/97:0.0152475056,SAJ298_E.sp_IRN:0.0054655187);

CONSENSUS TREE

Consensus tree is constructed from 1000 bootstrap trees

Log-likelihood of consensus tree: -2983.162967

Robinson-Foulds distance between ML tree and consensus tree: 4

Branches with support >0.000000% are kept (extended consensus)

Branch lengths are optimized by maximum likelihood on original alignment

Numbers in parentheses are bootstrap supports (%)

```
+--AR451-10_HQ982805_BATMAN_TUR
|
|              +---BJ001_E.granosus
|            +--| (71)
|            | |        +--KX537083_E.cinnaberinus_GER
|            | |      +--| (81)
|            | |      | +--OL352232.1_E.kollari_SAJ26
|            | |    +--| (99)
|            | |    | +--OL352226.1_E.kollari_SAJ128_CHI
|            | | +--| (94)
|            | | | +--SPICR045-10_JF886220_E.kollari_RUS
|            | +--| (80)
|            |   |      +--KY268507.1_E.moravicus_AUS
|            |   +--------| (100)
|            |           +--OL352218.1_E.moravicus_SAJ099
|          +--| (73)
|          | |                +--DQ973153_E.cinnaberinus_Ecinwest1_GER
|          | |          +-------| (99)
|          | |          |     | +--OL352235.1_E.cf._kollari_SAJ38
|          | |          |     +--| (92)
|          | |          |        +--SMNKARA08341_E.kollari_GER
|          | |        +--| (78)
|          | |        | | +-------OL352224.1_E.lavrosiae_SAJ12
|          | |        | +--| (70)
```

```
|          | |        |   +-----------OP434393.1_E.yukuni_YJL-2022b_20220914_CHI
|          | |      +--| (18)
|          | |      | |          +---------------------------FJ948997.1_E.sp._13_06_ISR
|          | |      | | +-----------| (96)
|          | |      | | |        |            +---FJ948999.1_E.walckenaeri_14_5_GRE
|          | |      | | |        +-----------------| (100)
|          | |      | | |                      +--OL352229.1_E.walckenaeri_SAJ14_TUR
|          | |      | +--| (51)
|          | |      |   | +--------FJ948998.1_E.cf._kollari_14_04_ITA
|          | |      |   +--| (90)
|          | |      |     +-------OL352228.1_E.sp._SAJ130_ITA
|          | |    +--| (40)
|          | |    | +-----------------SAJ106_E.agrinus_IRN
|          | | +--| (83)
|          | | | |          +--OP376824.1_E.da_20220728_CHI
|          | | | +---------------| (100)
|          | | |             +--SAJ319_E.sp._IRN
|          | +--| (62)
|          |   +---------SAJ320_E.sp._IRN
|        +--| (84)
|        | +---------SAJ321_E.sp._IRN
|      +--| (65)
|      | +--SAJ300_E.sp_BW_IRN
|   +--| (64)
|   | +--SAJ088_E.surena_IRN
| +--| (65)
| | |   +--KX443582.1_E.sandaliatus_DK
| | | +--| (98)
| | | | |   +--OL352220.1_E.hermani_SAJ103
| | | | | +--| (99)
| | | | | | +--SAJ175_E.hermani
```

```
| | | | +--| (81)
| | | |    +--OL352231.1_E.sandaliatus_SAJ17_NED
| | +--| (94)
| |    | +--OL352230.1_E.algarvensis_SAJ15
| |    +--| (100)
| |       +--SAJ286_E.sp_ARM
+--| (97)
| |       +--CaBOL_1018700
| |    +--| (39)
| |    | +--CaBOL_1018701
| | +--| (41)
| | | +--SAJ366_E.sp_ARM
| +--| (97)
|    +--SAJ367_E.sp_ARM
|
+--SAJ298_E.sp_IRN
```

Consensus tree in newick format:

(AR451-
10_HQ982805_BATMAN_TUR:0.0063866165,(((((((BJ001_E.granosus:0.0194459471,((((KX53708
3_E.cinnaberinus_GER:0.0019700810,OL352232.1_E.kollari_SAJ26:0.0026577484)81:0.001555
4310,OL352226.1_E.kollari_SAJ128_CHI:0.0052573421)99:0.0103076916,SPICR045-
10_JF886220_E.kollari_RUS:0.0093493352)94:0.0048911039,(KY268507.1_E.moravicus_AUS:0.
0000024859,OL352218.1_E.moravicus_SAJ099:0.0000024859)100:0.0393747751)80:0.009188
9157)71:0.0063482815,((((((DQ973153_E.cinnaberinus_Ecinwest1_GER:0.0000024892,(OL3522
35.1_E.cf._kollari_SAJ38:0.0000024859,SMNKARA08341_E.kollari_GER:0.0000024859)92:0.005
1101984)99:0.0364351691,(OL352224.1_E.lavrosiae_SAJ12:0.0367597168,OP434393.1_E.yuku
ni_YJL-
2022b_20220914_CHI:0.0528969593)70:0.0050645804)78:0.0141746126,((FJ948997.1_E.sp._1
3_06_ISR:0.1274242647,(FJ948999.1_E.walckenaeri_14_5_GRE:0.0200010893,OL352229.1_E.
walckenaeri_SAJ14_TUR:0.0157103995)100:0.0783533364)96:0.0528761338,(FJ948998.1_E.cf.
_kollari_14_04_ITA:0.0430679026,OL352228.1_E.sp._SAJ130_ITA:0.0373382002)90:0.01386515
28)51:0.0104326331)18:0.0000023336,SAJ106_E.agrinus_IRN:0.0811374734)40:0.0070773544,
(OP376824.1_E.da_20220728_CHI:0.0119766903,SAJ319_E.sp._IRN:0.0000021192)100:0.0710
942694)83:0.0113228219,SAJ320_E.sp._IRN:0.0449776354)62:0.0055731431)73:0.0080759726

,SAJ321_E.sp._IRN:0.0475780167)84:0.0121427787,SAJ300_E.sp_BW_IRN:0.0142484222)65:0.0054123388,SAJ088_E.surena_IRN:0.0071386305)64:0.0025875280,((KX443582.1_E.sandaliatus_DK:0.0000024859,((OL352220.1_E.hermani_SAJ103:0.0031164312,SAJ175_E.hermani:0.0032013734)99:0.0032321123,OL352231.1_E.sandaliatus_SAJ17_NED:0.0133523098)81:0.0028280743)98:0.0062975995,(OL352230.1_E.algarvensis_SAJ15:0.0014894535,SAJ286_E.sp_ARM:0.0031188455)100:0.0080805536)94:0.0032386815)65:0.0029226931,(((CaBOL_1018700:0.0014620894,CaBOL_1018701:0.0014618196)39:0.0000024859,SAJ366_E.sp_ARM:0.0000024859)41:0.0000024859,SAJ367_E.sp_ARM:0.0015244739)97:0.0095973838)97:0.0152331038,SAJ298_E.sp_IRN:0.0054603133);

ALISIM COMMAND

To simulate an alignment of the same length as the original alignment, using the tree and model parameters estimated from this analysis, you can use the following command:

```
--alisim simulated_MSA -t ERETG2.fas.treefile -m "TN{32.821,7.7279}+F{0.257875,0.127747,0.200135,0.414244}+I{0.605645}+G4{1.3515}" --length 658
```

To mimic the alignment used to produce this analysis, i.e. simulate an alignment of the same length as the original alignment, using the tree and model parameters estimated from this analysis *and* copying the same gap positions as the original alignment, you can use the following command:

```
iqtree -s ERETG2.fas --alisim mimicked_MSA
```

To simulate any number of alignments in either of the two commandlines above, use the --num-alignments options, for example mimic 100 alignments you would use the command line:

```
iqtree -s ERETG2.fas --alisim mimicked_MSA --num-alignments 100
```

For more information on using AliSim, please visit: www.iqtree.org/doc/AliSim

TIME STAMP

Date and time: Thu May 15 07:56:36 2025

Total CPU time used: 18.296875 seconds (0h:0m:18s)

Total wall-clock time used: 19.7645492 seconds (0h:0m:19s)
